# Development of a Low-Sodium Soaking Solution for Shrimp Processing Using Potassium Phosphates (KTPP and TKPP) and L-Lysine: Impacts on Quality and Water-Holding Capacity

**DOI:** 10.3390/foods15173063

**Published:** 2026-08-29

**Authors:** Teerapat Monvises, Khanittha Chinarak, Sasina Sanprasert, Anurak Uchuwittayakul, Piyangkun Lueangjaroenkit, Samart Sai-ut, Jaksuma Pongsetkul, Saroat Rawdkuen, Passakorn Kingwascharapong

**Affiliations:** 1 Department of Fishery Product, Faculty of Fisheries, Kasetsart University, Bangkok 10900, Thailand; teerapat.monv@ku.th (T.M.); khanittha.ch@ku.th (K.C.); sasina.sanp@ku.th (S.S.); 2 Department of Aquaculture, Faculty of Fisheries, Kasetsart University, Bangkok 10900, Thailand; ffisarb@ku.ac.th; 3 Department of Microbiology, Faculty of Science, Kasetsart University, Bangkok 10900, Thailand; piyangkun.lu@ku.th; 4 Department of Food Science, Faculty of Science, Burapha University, Chonburi 20131, Thailand; samarts@go.buu.ac.th; 5 School of Animal Technology and Innovation, Institute of Agricultural Technology, Suranaree University of Technology, Nakhon Ratchasima 30000, Thailand; jaksuma@sut.ac.th; 6 Unit of Innovative Food Packaging and Biomaterials, School of Agro-Industry, Mae Fah Luang University, Chiang Rai 57100, Thailand; saroat@mfu.ac.th

**Keywords:** Pacific white shrimp, sodium reduction, soaking, potassium tripolyphosphate, tetrapotassium pyrophosphate, water-holding capacity, consumer acceptance

## Abstract

A low-sodium soaking solution for Pacific white shrimp was developed by partially substituting sodium tripolyphosphate (STPP) with potassium tripolyphosphate (KTPP) or tetrapotassium pyrophosphate (TKPP) (0.5–2.5%, *w*/*v*) in combination with L-lysine (2%, *w*/*v*). The effects on physicochemical properties, microstructure, and consumer acceptance were evaluated in comparison with a non-soaked control and a positive control (3% STPP, *w*/*v*). The results indicated that partial substitution of STPP with KTPP or TKPP could be effectively achieved while maintaining processing yield, color, and water-holding capacity (WHC) comparable to the positive control. Scanning electron microscopy revealed a more relaxed and expanded muscle structure, indicating enhanced water retention and reduced shear force. However, no significant differences in shear force were observed between the substitution treatments and the positive control. Substitution with KTPP or TKPP significantly reduced phosphate content and the Na/K ratio. Sodium levels decreased by 23.5–47.0% with KTPP and 28.8–59.5% with TKPP, while potassium content increased in the treated shrimp. This substitution did not adversely affect consumer acceptance, with improved taste characteristics, particularly enhanced saltiness and umami, detected by electronic-tongue analysis. Overall, replacing STPP with KTPP or TKPP shows potential as a strategy for developing reduced-sodium shrimp products while maintaining product quality.

## 1. Introduction

Nowadays, health concerns regarding excessive sodium consumption are a growing global issue, as high sodium intake is directly linked to an increased risk of Non-Communicable Diseases (NCDs), particularly cardiovascular and kidney diseases [1,2]. The World Health Organization (WHO) recommends a daily sodium limit of 2000 mg [3]. However, in Thailand, the mean sodium intake is approximately 3636 mg per day, nearly double the recommended threshold [4]. A significant portion of this intake originates from processed foods, where sodium can contribute up to 20% of daily consumption [5].

Reducing sodium in processed foods remains a formidable challenge for the food industry. Beyond its flavor-enhancing properties, sodium plays a pivotal role in the extraction of myofibrillar proteins, which are indispensable for effective gelation and textural integrity [6,7]. Furthermore, sodium chloride contributes to water-holding capacity, thereby minimizing cooking loss and ensuring a tender, juicy product [8]. Consequently, the reduction of sodium without effective functional substitutes leads to detrimental impacts on overall product quality and sensory acceptability.

Pacific white shrimp (PWS; *Litopenaeus vannamei*) is a widely popular seafood due to its high-quality protein, complete essential amino acid profile, and beneficial unsaturated fatty acids [9,10]. Furthermore, due to its rapid growth rate and robust environmental adaptability, PWS represents a primary aquatic export for Thailand in both fresh and frozen forms [11].

In the seafood industry, sodium phosphates, particularly sodium tripolyphosphate (STPP), are extensively utilized as functional additives to enhance water-holding capacity (WHC) and improve sensory attributes [12]. However, the application of STPP contributes to elevated sodium levels in the final product, which may pose health risks if consumed excessively [6,13]. Consequently, substituting sodium-based phosphates with potassium-based alternatives, such as potassium tripolyphosphate (KTPP) or tetrapotassium pyrophosphate (TKPP), represents a promising strategy. Potassium phosphates have been shown to maintain functional properties, including moisture retention and gelation, at levels comparable to their sodium counterparts [14]. This functionality is primarily attributed to the synergistic effect of increasing ionic strength and shifting the pH away from the isoelectric point (pI), thereby inducing the swelling of muscle fibers [15]. However, it is recognized that high inclusion levels of potassium salts can impart objectionable off-flavors, such as bitterness and metallic notes [16]. Furthermore, the incorporation of basic amino acids, such as L-lysine, has emerged as a bio-based approach to enhance gelation and effectively reduce sodium content in shrimp products [17,18]. In this study, L-lysine was incorporated as a constant baseline component across all treatments to compensate for potential losses in water-holding capacity (WHC) during STPP reduction, supporting an integrated low-sodium formulation strategy while maintaining product quality.

The objective of this study is to investigate the effects of substituting STPP with varying proportions of KTPP and TKPP, in combination with L-lysine 2% (*w*/*v*), on the physicochemical properties, microstructure, and consumer acceptance of PWS. This research aims to develop an innovative low-sodium soaking solution that preserves product quality while promoting health benefits for consumers.

## 2. Materials and Methods

### 2.1. Chemical Reagents

Sodium tripolyphosphate (STPP), potassium tripolyphosphate (KTPP) and tetrapotassium pyrophosphate (TKPP) were obtained from Aditya Birla Chemicals (Thailand) Ltd., Samut Prakan, Thailand. L-lysine was purchased from Sigma Chemical Co. (St. Louis, MO, USA). Sodium chloride (NaCl) and potassium chloride (KCl) were from Nature Lab Co., Ltd., Bangkok, Thailand. Sodium hydroxide (NaOH) and hydrochloric acid (HCl) were the products of Merck (KGaA, 64271 Darmstadt, Germany). All chemicals used are analytical grade.

### 2.2. Sample Preparation

Peeled and deveined tail-on PWS (*Litopenaeus vannamei*), with a size of 50–55 shrimp/kg, were purchased from a commercial farm in Samut Prakan Province, Thailand. To minimize biological raw material variability, a single uniform lot of fresh PWS was used for the entire experiment. The fresh PWS were iced and transported to the Department of Fishery Products, Faculty of Fisheries, Kasetsart University (Bangkok, Thailand), within 1 h. Upon arrival, the fresh PWS were rinsed under running potable water and de-tailed to obtain peeled and deveined tail-less PWS. This method has been reviewed and approved by the Kasetsart University Institutional Animal Care and Use Committee (U1-10911-2566).

The prepared shrimp were then immersed in various soaking solutions, as specified in Table 1, at a shrimp-to-solution ratio of 1:2 (*w*/*v*), corresponding to 100 g of shrimp per treatment replicate [12,17,18]. For each treatment, three soaking beakers were prepared separately, with fresh soaking solution prepared for each beaker. Each beaker contained approximately 100 g of shrimp and 200 mL of soaking solution. Thus, three beakers were used as the experimental units for each treatment (*n* = 3). The soaking process was conducted for 60 min at 4 °C, with continuous stirring during the first 30 min. After treatment, the samples were removed from the soaking solution, placed in a strainer, and allowed to drain for approximately 5 min to remove excess surface moisture. The treated shrimp were then steamed using a stainless-steel steamer over boiling water until the core temperature reached 90 °C (requiring approximately 270 s). The cooked shrimp were immediately transferred to a strainer and cooled in iced water. The strainer was then lifted, and the shrimp were allowed to drain for approximately 5 min to remove excess water before being packed in polyethylene (PE) bags (BKK Packaging, Bangkok, Thailand) and stored in ice during subsequent analyses.

### 2.3. Determination of pH

The pH values of both the soaking solutions and the shrimp muscle were determined using a digital pH meter (model MM-60R, TOA DKK, Japan) equipped with a glass combination pH electrode. For the soaking solutions, pH was measured directly for each of the three soaking beakers (*n* = 3). For shrimp muscle, samples were randomly selected separately from each soaking beaker, and one value was obtained for each beaker for statistical analysis (*n* = 3). The pH of shrimp muscle was measured following the modified procedure of [19]. The shrimp (2 g) were homogenized with 10 mL of distilled water at 1000 rpm for 1 min. The homogenized sample was allowed to equilibrate at room temperature for 5 min prior to measurement. For soaking solutions, the pH was measured directly without dilution.

### 2.4. Determination of Weight Gain, Cooking Loss and Cooking Yield

The weight of the shrimp was recorded before ($W_{1}$) and after the soaking treatment ($W_{2}$). Following the immersion process, the samples were removed from the soaking solution and drained for approximately 5 min as described in Section 2.2 before weighing. The weight gain percentage was calculated using Equation (1), as described by Chantarasuwan et al. [20].

(1) $$\mathrm{Weight}\;\mathrm{Gain}\;(\%)=(W_{2}-W_{1})/W_{1}\times 100$$

where $W_{1}$ and $W_{2}$ represent the weights of the shrimp before and after soaking, respectively.

Cooking loss and cooking yield were evaluated to monitor weight changes throughout the processing stages. The soaked shrimp samples ($W_{2}$) were steamed and cooled as described in Section 2.2, followed by draining for approximately 5 min before being weighed ($W_{3}$).

Cooking loss was calculated using Equation (2) based on the weight difference between the soaked ($W_{2}$) and steamed ($W_{3}$) weights, following the method described by He et al. [21].

(2) $$\mathrm{Cooking}\;\mathrm{Loss}\;(\%)=(W_{2}-W_{3})/W_{2}\times 100$$

Cooking yield was calculated using Equation (3). The initial weight before soaking was determined ($W_{1}$) relative to the final cooked weight ($W_{3}$), according to the protocol by Chantarasuwan, Benjakul and Visessanguan [20].

(3) $$\mathrm{Cooking}\;\mathrm{Yield}\;(\%)=(W_{3}/W_{1})\times 100$$

For each treatment, three separately prepared soaking beakers were used as independent experimental units. Measurements of weight gain, cooking loss, and cooking yield were obtained separately for each beaker, and the resulting three beaker-level values were used for statistical analysis (*n* = 3).

### 2.5. Determination of Water-Holding Capacity (WHC)

WHC was evaluated through free drip loss and expressible drip, adapted from the methods of [22]. Samples were randomly selected separately from each of the three soaking beakers for each treatment. Measurements were performed for each beaker, and one beaker-level value was obtained for each treatment replicate (*n* = 3). For free drip, shrimp muscle from the second abdominal segment was weighed ($M_{1}$), placed between two layers of filter paper (Whatman No. 1), and stored at 4 °C for 2 h before being reweighed ($M_{2}$). For expressible drip, samples were placed between filter papers (two layers on top and three layers on the bottom) and subjected to compression using a texture analyzer (TA.XTplusC, Stable Micro Systems, Surrey, UK) equipped with a P/50 cylindrical probe. The instrument settings were as follows: a pre-test speed of 1.0 mm/s, a test speed of 0.5 mm/s, and a post-test speed of 10.0 mm/s. A compression force of 1000 g was applied and held constant for 2 min. Following compression, the final weight of the sample was recorded as $M_{3}$. Free drip and expressible drip were calculated using Equations (4) and (5), respectively:

(4) $$\mathrm{Free}\;\mathrm{Drip}\;(\%)\;=(M_{1}-M_{2})/M_{1}\times 100$$

(5) $$\mathrm{Expressible}\;\mathrm{Drip}\;(\%)=(M_{1}-M_{3})/M_{1}\times 100$$

### 2.6. Determination of Color

The color of the cooked shrimp muscle was determined at the second abdominal segment using a colorimeter (UltraScan VIS colorimeter, HunterLab, Reston, VA, USA) with *d*/8° geometry, D65 illuminant, 10° observer, and a 25-mm LAV port. Ten individual cooked shrimp in total were randomly selected across the three soaking beakers for each treatment to account for surface color variability. The color measurements of shrimp from each beaker were averaged to obtain one beaker-level value, and the three beaker-level values were used for statistical analysis (*n* = 3). The L* values evaluate the lightness of samples, the a* values measure the degree of redness to greenness, and the b* values evaluate the degree of yellowness to blueness.

### 2.7. Determination of Shear Force

The shear force of cooked shrimp was determined using a texture analyzer (TA.XTplusC, Stable Micro Systems, Surrey, UK) equipped with a Warner–Bratzler shear blade (HDP/WBS), following the method described by [23]. Ten individual cooked shrimp in total were randomly selected across the three soaking beakers for each treatment to account for surface color variability. The color measurements of shrimp from each beaker were averaged to obtain one beaker-level value, and the three beaker-level values were used for statistical analysis (*n* = 3). The instrument was operated at a cross-head speed of 10 mm/s with a 50 kg load cell. Shear force was measured perpendicular to the orientation of the muscle fibers in the second abdominal segment.

### 2.8. Microstructure

Microstructure was analyzed according to the method described by Rattanasatheirn et al. [24]. Samples were immersed in liquid nitrogen and allowed to stand at room temperature for 5 min. Thereafter, the samples were cut into cubes (4 × 4 × 4 mm) using a sharp razor blade. The specimens were fixed in 2.5% (*v*/*v*) glutaraldehyde in 0.2 M phosphate buffer (pH 7.2) at room temperature for 2 h. All specimens were washed three times with deionized water for 15 min each and dehydrated through a graded ethanol series of 20, 50, 60, 70, 80, 90, and 100% for 15 min at each concentration. The specimens were then coated with gold using a sputter coater (Quorum Q150 RES). The microstructure was visualized using a scanning electron microscope (Quanta 450, FEI Company, Brno, OR, Czech Republic). Magnifications of 10,000× and 500× were used for the longitudinal and cross sections, respectively.

### 2.9. Determination of Total Phosphate Content

The total phosphate content of the cooked shrimp was determined spectrophotometrically using a spectrophotometer (Spectro Evolution 300, Thermo Scientific, Madison, WI, USA), following the AOAC official colorimetric method with slight modifications. Three independent analytical replicates were performed (*n* = 3). For sample preparation, the same weight of cooked shrimp (10 g) per analytical replicate was homogenized with 20 mL of 10% (*w*/*v*) trichloroacetic acid (TCA) and stirred for 5 min. The homogenate was filtered through filter paper (Whatman No. 1). The pH of the filtrate was adjusted to 4.5–5.0 using 1 N NaOH, and the final volume was made up to 50 mL with distilled water. For color development, the extract (1 mL) was diluted to 25 mL with distilled water. Subsequently, the diluted extract was mixed with distilled water (1:9, *v*/*v*), and 1 mL of 1.5% (*w*/*v*) ammonium molybdate solution was added. The mixture was heated in a water bath (95 °C) for 10 min, followed by adding 1 mL of 1% (*w*/*v*) hydrazine sulfate solution. After reheating for 10 min and cooling in cold water for 20 min, the absorbance was measured at 830 nm. A calibration curve was created with potassium dihydrogen phosphate (0–25 µg/mL) as a standard. The phosphate content was expressed as mg/kg.

### 2.10. Determination of Sodium and Potassium Contents

The sodium (Na) and potassium (K) contents of the cooked shrimp samples were determined according to the method described by Chueadoem et al. [25]. Three independent analytical replicates were performed (*n* = 3), with each replicate measured in technical triplicate and the triplicate measurements averaged. Briefly, cooked shrimp (0.5 g) was accurately weighed into digestion vessels and treated with 8 mL of 69% nitric acid (HNO_3_) and 2 mL of hydrogen peroxide (H_2_O_2_). The samples were allowed to pre-digest at room temperature for 10 min before being completely mineralized using a microwave digestion system. Following digestion, the resulting solutions were cooled to ambient temperature and diluted to a final volume of 50 mL with deionized water. The quantitative determination of Na and K was performed using an Atomic-Absorption Spectrophotometer (AAS; model ContrAA 800, Analytik Jena, Germany). Mineral concentrations were calculated based on calibration curves constructed from standard solutions of known concentrations. Measurements were conducted at specific wavelengths of 589.0 nm for Na and 766.5 nm for K. The results were averaged and reported as mg/kg of cooked shrimp.

### 2.11. Electronic-Tongue (E-Tongue) Analysis

The taste profile of the cooked shrimp was determined using an electronic-tongue (E-tongue) system (TS-6000A, Insent, Atsugi, Japan). The analysis was conducted according to the method described by [26], with slight modifications. Eight sensors (sour, bitter, astringent, umami, salty, bitter_aft, astringent_aft and umami_aft) were used in this study and the reference electrodes were activated before analysis. Cooked shrimp (60 g) was mixed with 240 mL of distilled water and homogenized for 1 min before being centrifuged at 3000 rpm for 10 min. The supernatants were filtrated and subsequently injected into the electronic-tongue system for analysis. The data were analyzed using the Taste Sensing System Analysis Application.

### 2.12. Preliminary Consumer Test

A preliminary consumer test of cooked shrimp was conducted using thirty consumers (15 males and 15 females, aged 18–30 years) recruited from students and staff of the Department of Fishery Products, Faculty of Fisheries, Kasetsart University, Bangkok, Thailand. All consumers had successfully completed academic coursework and practical training in sensory evaluation of aquatic food products, specifically seafood quality assessment. Participants were regular consumers of shrimp (consuming shrimp at least once a week) and were completely blinded to the research objectives and treatment formulations.

Evaluation sessions were conducted in individual sensory booths under balanced white light. Each consumer evaluated 12 cooked shrimp samples, with one whole cooked shrimp representing each treatment. Samples were served in coded transparent tasting cups labeled with three-digit random numbers. To avoid sensory fatigue and order bias, samples were presented simultaneously in a randomized, counterbalanced serving order. Using a 9-point hedonic scale (1 = dislike extremely, 5 = neither like nor dislike, 9 = like extremely), consumers rated appearance, color, flavor, texture, and overall acceptability. Participants were instructed to rinse their mouths thoroughly with room-temperature drinking water during a 2-min inter-sample interval. This consumer test protocol was reviewed and approved by the Kasetsart University Research Ethics Committee (KUREC-HSR68/041).

### 2.13. Statistical Analysis

All soaking treatments and processing procedures were performed in triplicate using three separately prepared soaking beakers as independent experimental units (*n* = 3). The number of replicates used for each parameter is specified in the corresponding methods section. A completely randomized design (CRD) was employed for physicochemical parameters, whereas a randomized complete block design (RCBD) was used for the preliminary consumer test. For the physicochemical analyses, the KTPP- and TKPP-containing formulations were treated as predefined complete treatment formulations because the objective of the study was to compare specific STPP-replacement formulations rather than to independently estimate the effects of phosphate type, substitution level, or their interaction. For the preliminary consumer test, each consumer served as a block because the same consumers evaluated multiple treatment samples, thereby accounting for variation among consumers.

The substitution concentrations (0.5–2.5%) were treated as predefined formulation levels rather than as a continuous predictor because the study was designed to compare specific replacement formulations rather than to develop a regression model or determine a mathematical optimum. Data were confirmed to meet ANOVA assumptions prior to statistical testing. Analysis of variance (One-Way ANOVA) was performed using the SPSS software package (SPSS 23.0 for Windows, SPSS Inc., Chicago, IL, USA). Duncan’s multiple range test was used to assess differences between treatment means at *p* < 0.05.

## 3. Results and Discussion

### 3.1. pH of Solution and Shrimp Meat

As shown in Table 2, the pH of the positive control was 7.99, while the pH values of KTPP solutions ranged from 7.87 to 8.00, and those of TKPP solutions ranged from 8.17 to 8.51. With increasing KTPP concentration, a slight decrease in pH was observed, which may be attributed to the relatively lower alkalinity of KTPP compared with TKPP, resulting in a lower contribution to the basicity of the solution [16,27]. In contrast, increasing the concentration of TKPP led to a progressive increase in pH, likely due to its greater alkalinity and the acid–base behavior of the pyrophosphate ion in aqueous solution, thereby enhancing the overall alkalinity of the system [28]. This trend is consistent with the intrinsic chemical properties of the individual phosphate compounds and their distinct ionic dissociation behaviors [29].

For both raw and cooked shrimp, the pH of the treated samples was higher than that of the control, which may be attributed to the influence of the soaking solutions. The alkaline conditions of the soaking solutions may shift the muscle protein system further away from its isoelectric point (pI ≈ 5.0) during soaking, thereby increasing the net negative charge on protein molecules and enhancing electrostatic repulsion [20,28]. This electrostatic repulsion promotes myofibrillar swelling and expansion of interfilament spaces, increasing the capillary volume available for water entrapment [6,20,30]. The decrease in pH of the soaking solutions after immersion may be explained by diffusion of alkaline ions into the shrimp tissue and subsequent ion exchange with functional groups of myofibrillar proteins, as previously reported by Chantarasuwan, Benjakul and Visessanguan [20]. After cooking, the pH of shrimp muscle was higher than that of the raw soaked samples, which may be attributed to heat-induced protein denaturation and aggregation, leading to structural rearrangement and exposure of charged groups within the protein matrix [21]. In addition, the formation and accumulation of basic volatile nitrogenous compounds, such as ammonia and amines generated from thermal degradation of proteins and peptides, may further contribute to the increased pH in cooked tissues [31].

### 3.2. Weight Gain, Cooking Loss and Cooking Yield

**Figure 1:**
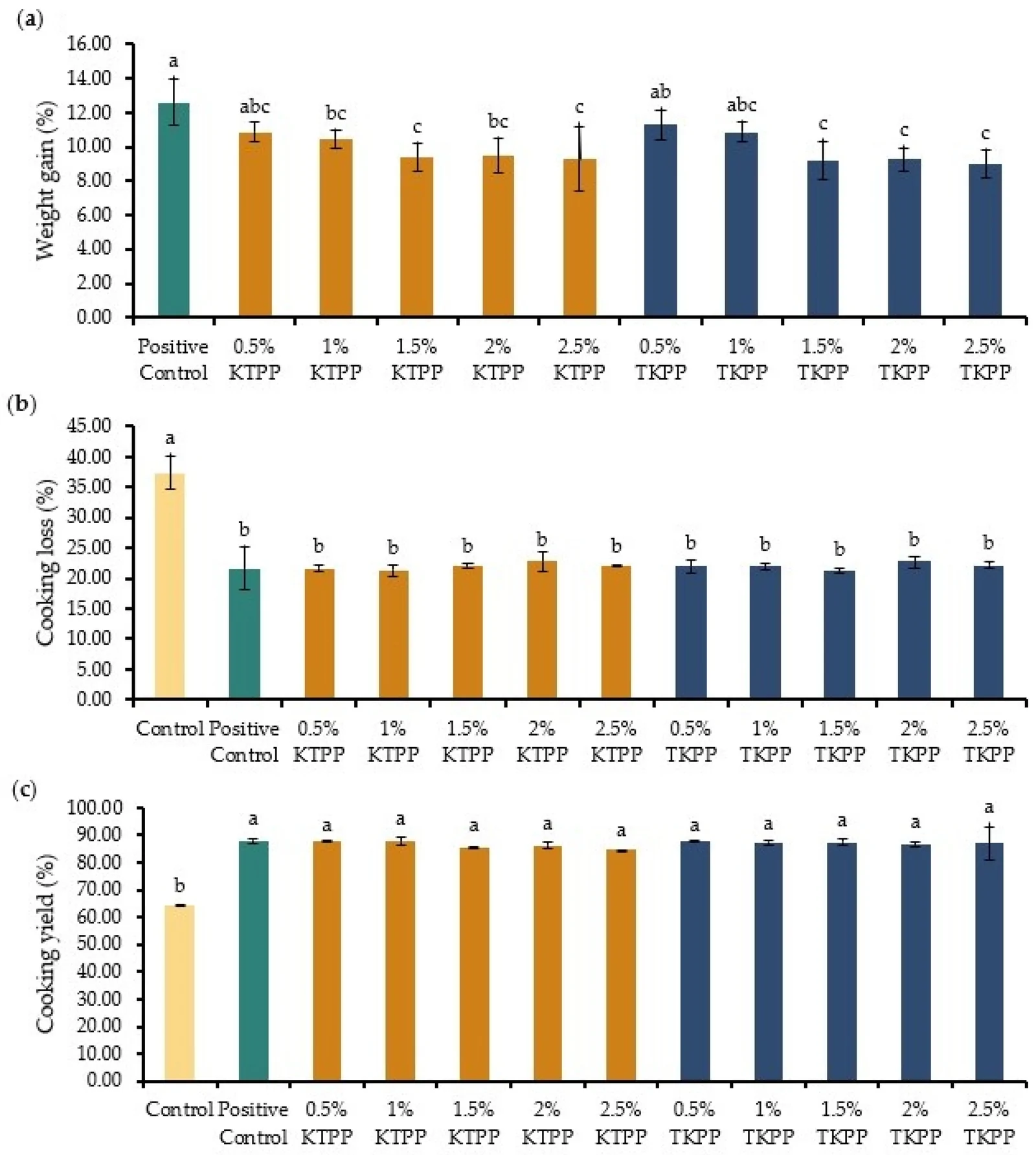
Weight gain (**a**), cooking loss (**b**), and cooking yield (**c**) of shrimp treated with different soaking solutions. The positive control consisted of 2.5% NaCl, 3.0% sodium tripolyphosphate (STPP), and 2.0% L-lysine. In the KTPP and TKPP treatments, STPP was partially replaced with potassium tripolyphosphate (KTPP) or tetrapotassium pyrophosphate (TKPP) at levels ranging from 0.5 to 2.5% (*w*/*v*). Different lowercase letters indicate differences (*p* < 0.05).

Weight gain, cooking loss, and cooking yield are key indicators of the effectiveness of soaking treatments in improving the water-holding capacity of PWS, reflecting moisture retention, processing yield, and product quality [30]. Replacing STPP with varying proportions of KTPP and TKPP combined with L-lysine affected weight gain, while cooking loss and cooking yield remained comparable to those of the positive control (Figure 1).

Among the treatments, soaking in 0.5% KTPP, 0.5% TKPP, or 1% TKPP resulted in weight gain comparable to that of the positive control (STPP) (Figure 1a), with no significant differences. However, weight gain gradually decreased with increasing proportions of KTPP or TKPP relative to the positive control (*p* < 0.05). This trend may be attributed to the lower effectiveness of potassium ions compared to sodium ions in promoting myofibrillar protein solubilization and swelling, which are essential for enhancing water uptake [32]. In addition, increasing the substitution level of STPP with KTPP or TKPP may alter the overall ionic strength and phosphate functionality of the soaking solution, thereby reducing the efficiency of actomyosin dissociation and limiting the availability of water-binding sites [28,29,33]. Furthermore, increasing substitution levels of KTPP or TKPP may induce osmotic effects or partial structural destabilization of muscle proteins, leading to reduced water retention and, consequently, lower weight gain relative to the positive control. Although weight gain decreased at higher substitution levels of KTPP or TKPP relative to the positive control, all blended phosphate treatments showed cooking loss and cooking yield comparable to those of the positive control (Figure 1b,c), indicating that partial replacement of STPP with KTPP or TKPP did not compromise water retention or processing yield during thermal processing. This maintenance of water retention and processing yield may be attributed to the synergistic effects of NaCl, phosphates, and L-lysine, which collectively enhance the water-holding stability of shrimp muscle during soaking [17,18,30]. NaCl increases ionic strength and promotes actomyosin dissociation, facilitating protein solubilization [20,34]. Meanwhile, phosphates elevate pH, increase the net negative charge of muscle proteins, and expand the myofibrillar lattice, thereby enhancing electrostatic repulsion and improving water immobilization within the muscle structure [35]. In addition, L-lysine, as a polar amino acid, may interact with muscle proteins through ionic and hydrogen bonding, contributing to protein unfolding and increasing the availability of water-binding sites, which further enhances water retention [36]. Therefore, partial substitution of STPP with KTPP or TKPP combined with L-lysine effectively preserved processing yield and water retention in PWS, highlighting its potential as a functional reduced-sodium soaking system.

### 3.3. Water-Holding Capacity (WHC)

**Figure 2:**
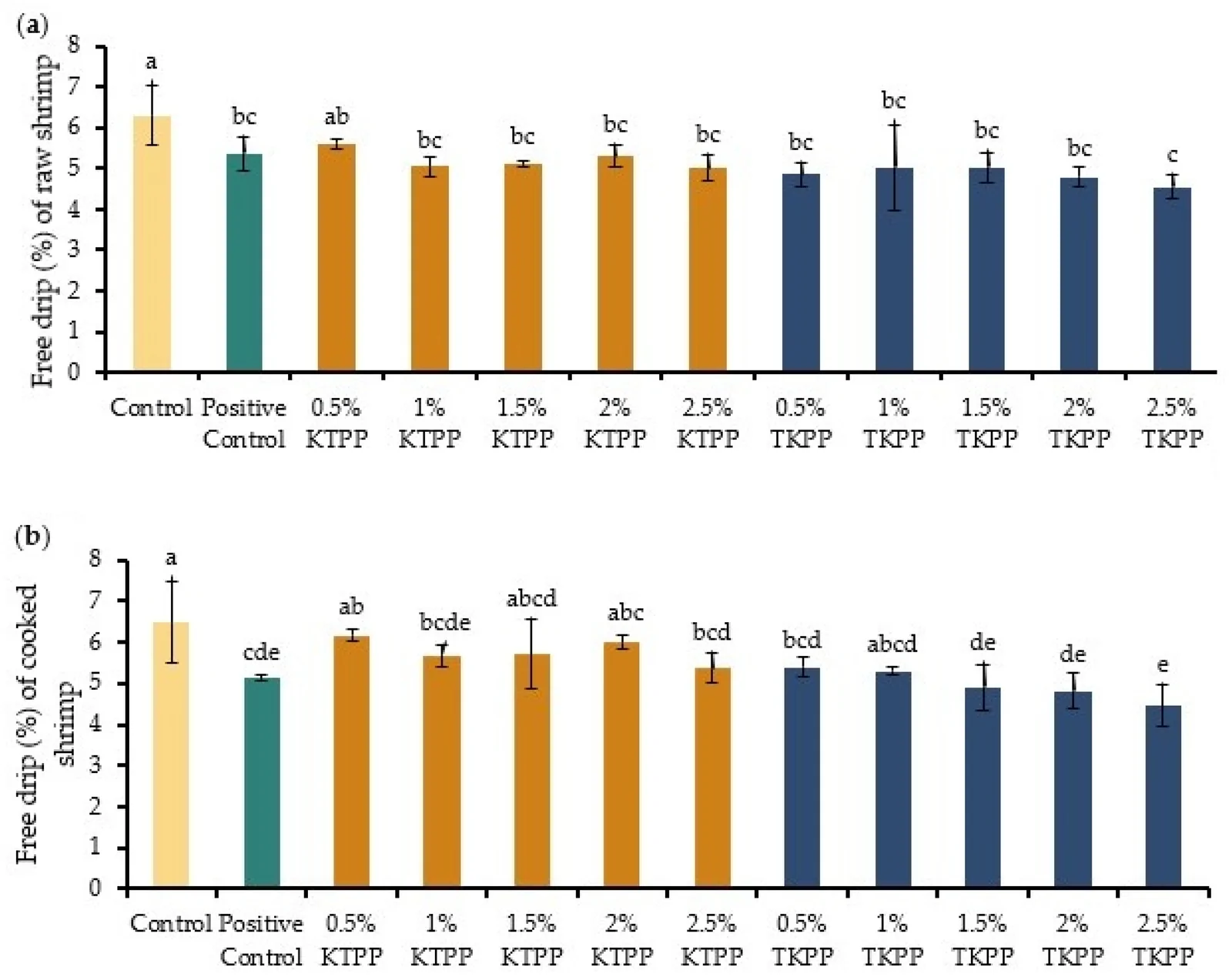
Free drip of raw (**a**) and cooked shrimp (**b**) treated with different soaking solutions. The positive control consisted of 2.5% NaCl, 3.0% sodium tripolyphosphate (STPP), and 2.0% L-lysine. In the potassium tripolyphosphate (KTPP) or tetrapotassium pyrophosphate (TKPP) treatments, STPP was partially replaced with KTPP or TKPP at levels ranging from 0.5 to 2.5% (*w*/*v*). Different lowercase letters indicate difference (*p* < 0.05).

**Figure 3:**
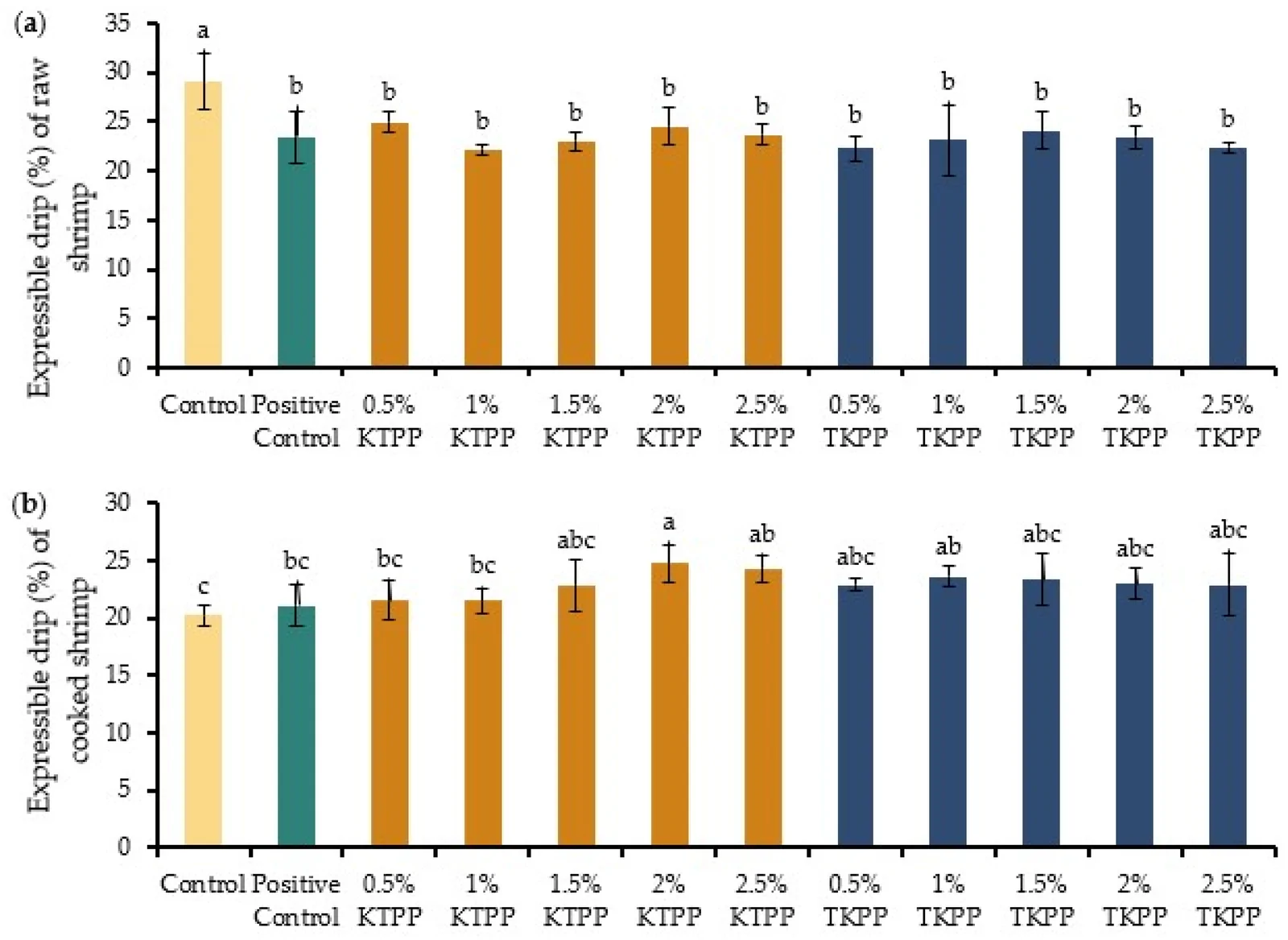
Expressible drip of raw (**a**) and cooked shrimp (**b**) treated with different soaking solutions. The positive control consisted of 2.5% NaCl, 3.0% sodium tripolyphosphate (STPP), and 2.0% L-lysine. In the potassium tripolyphosphate (KTPP) or tetrapotassium pyrophosphate (TKPP) treatments, STPP was partially replaced with KTPP or TKPP at levels ranging from 0.5 to 2.5% (*w*/*v*). Different lowercase letters indicate difference (*p* < 0.05).

Water-holding capacity (WHC) is a critical parameter for evaluating the textural quality and water retention of aquatic products, as fresh shrimp usually contain approximately 75% moisture [37,38]. The WHC of raw and cooked shrimp muscle, as reflected by the percentages of free drip and expressible drip, is presented in Figure 2. The free drip of raw shrimp decreased following soaking treatments (Figure 2a). Although no significant differences were observed among the treated groups, all treatments except the 0.5% KTPP treatment exhibited significantly lower free drip compared to the control, indicating improved water retention in the muscle matrix. A similar trend was observed in cooked shrimp (Figure 2b), where all treatments showed lower free drip compared to the control. Regarding expressible drip (Figure 3), raw shrimp immersed in all treatment solutions exhibited significantly lower values than the non-soaked control (Figure 3a), indicating enhanced water retention. However, varying ratios of KTPP or TKPP had no significant effect compared to the positive control, suggesting comparable functionality among the phosphate blends. For cooked shrimp (Figure 3b), the control exhibited the lowest expressible drip compared to all treatments. This result may be attributed to the limited water-holding capacity of the control sample, as evident from its high cooking loss (Figure 1b) and low cooking yield (Figure 1c), leading to substantial moisture loss during thermal processing. Consequently, less water remained within the muscle structure to be released upon compression, resulting in lower expressible drip values. In contrast, the alkaline soaking conditions may have contributed to greater water retention during heating. As a result, a greater amount of immobilized water remained within the muscle matrix and was subsequently released during compression, leading to higher expressible drip values compared to the control. Thus, the blending of phosphates may promote the water-holding capacity of shrimp muscle under the applied soaking conditions.

### 3.4. Color

**Figure 4:**
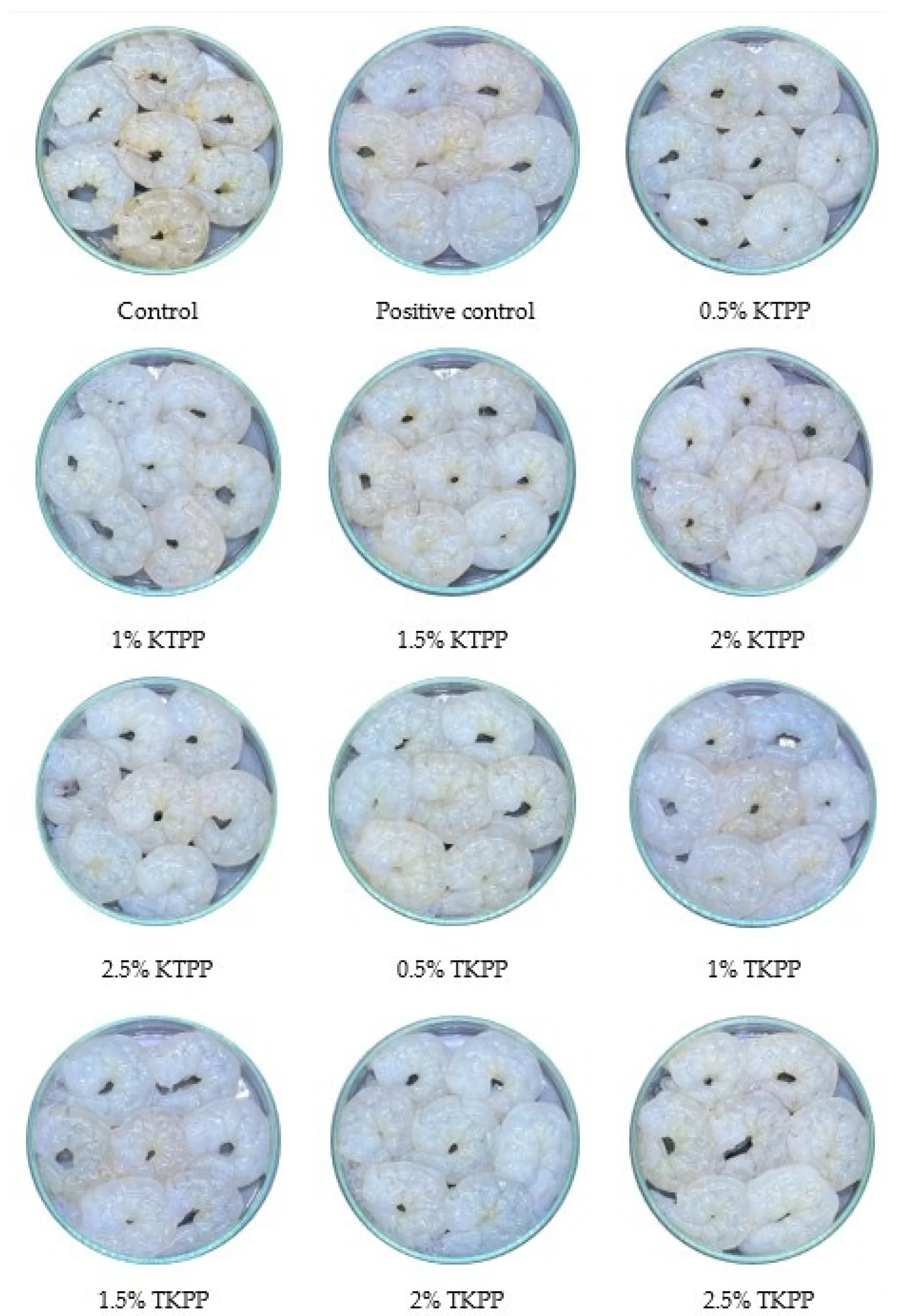
Images of raw shrimp treated with different soaking solutions. The positive control consisted of 2.5% NaCl, 3.0% sodium tripolyphosphate (STPP), and 2.0% L-lysine. In the KTPP and TKPP treatments, STPP was partially replaced with potassium tripolyphosphate (KTPP) or tetrapotassium pyrophosphate (TKPP) at levels ranging from 0.5 to 2.5% (*w*/*v*).

Color is a fundamental sensory attribute in food evaluation and plays an important role in shaping consumers’ perceptions, preferences, and food choices [39]. The color of different shrimp is shown in Table 3. For the raw shrimp, the L* values of the control were significantly lower than that of the treated samples, which may be attributed to the swelling of the myofibrillar structure and improved water retention induced by the soaking treatments, resulting in enhanced light scattering and reflection from the shrimp surface. However, no significant differences were observed between the positive control and the treated samples (Figure 4), likely due to the comparable effectiveness of phosphate-based treatments in promoting protein hydration and structural expansion.

**Figure 5:**
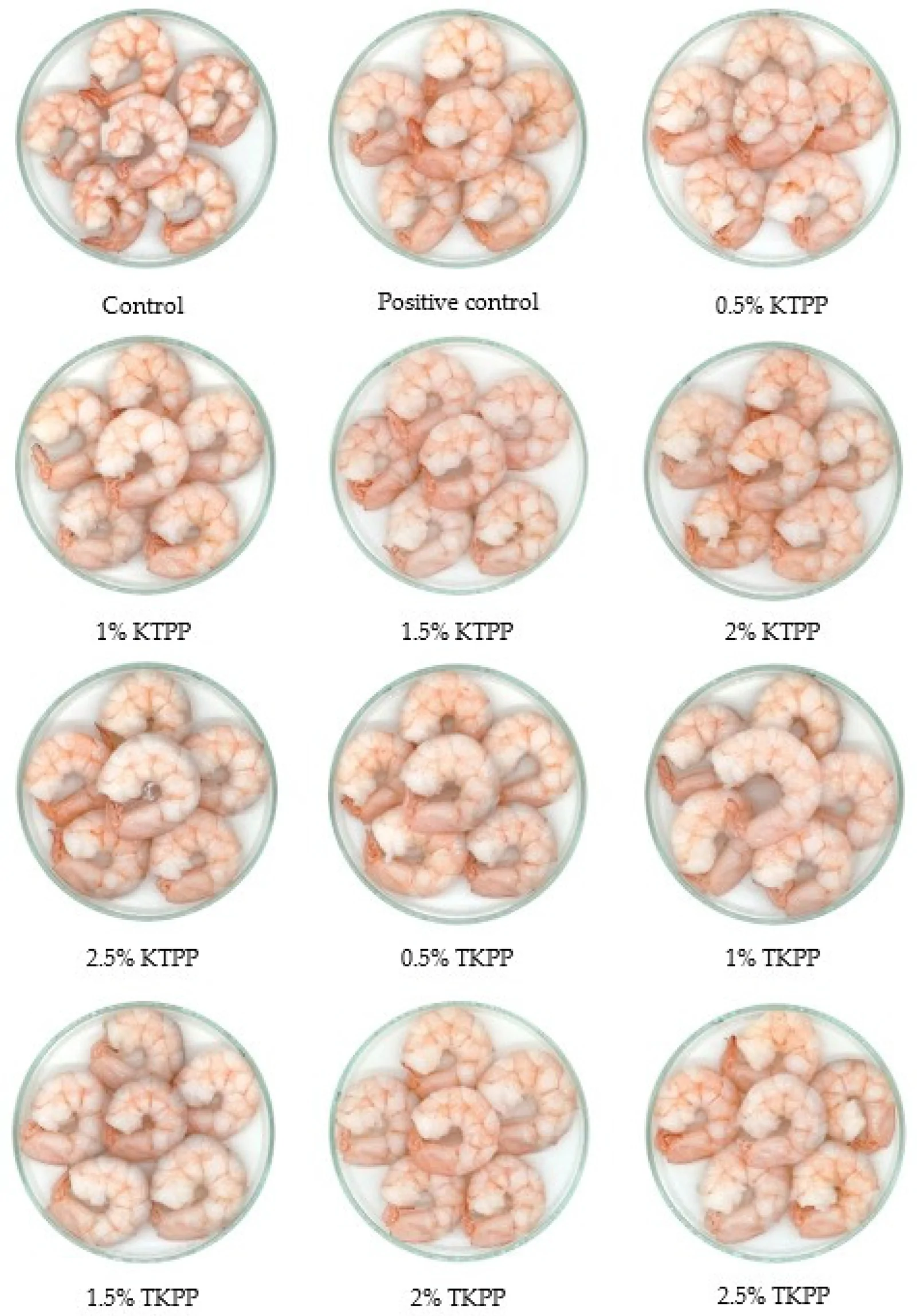
Images of cooked shrimp treated with different soaking solutions. The positive control consisted of 2.5% NaCl, 3.0% sodium tripolyphosphate (STPP), and 2.0% L-lysine. In the KTPP and TKPP treatments, STPP was partially replaced with potassium tripolyphosphate (KTPP) or tetrapotassium pyrophosphate (TKPP) at levels ranging from 0.5 to 2.5% (*w*/*v*).

For the cooked shrimp, slight variations in L* values were observed among treatments; however, the values remained within a narrow range (69–72), indicating similar lightness across samples. Likewise, the redness (a*) and yellowness (b*) values were within the ranges of 5–7 and 10–14, respectively, suggesting comparable color characteristics among treatments (Figure 5). Such variation may be attributed to the inherent biological variability of shrimp, including differences in muscle composition and microstructure. Notably, the reddish-orange coloration of boiled shrimp is widely recognized as an important quality attribute, as consumers generally prefer a bright and vivid appearance [17,21,30]. This coloration originates from the carotenoid astaxanthin, which is typically bound to carotenoproteins in muscle tissue; during thermal processing, these complexes denature, releasing free astaxanthin and resulting in the development of a reddish color [40]. Therefore, replacing STPP with KTPP or TKPP in combination with L-lysine in the soaking solution did not adversely affect the characteristic reddish coloration of cooked shrimp, which remained comparable to that of the non-soaked control.

### 3.5. Shear Force

**Figure 6:**
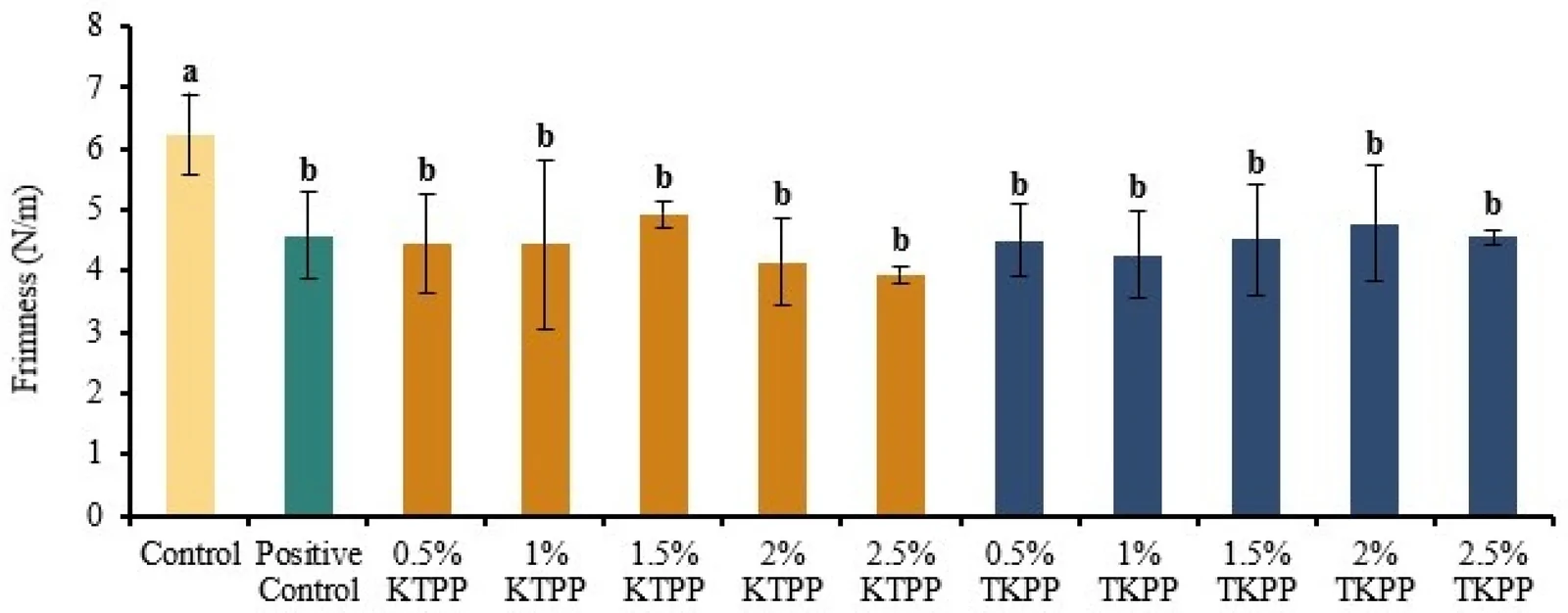
Shear force (firmness) of cooked shrimp treated with different soaking solutions. The positive control consisted of 2.5% NaCl, 3.0% sodium tripolyphosphate (STPP), and 2.0% L-lysine. In the KTPP and TKPP treatments, STPP was partially replaced with potassium tripolyphosphate (KTPP) or tetrapotassium pyrophosphate (TKPP) at levels ranging from 0.5 to 2.5% (*w*/*v*). Different lowercase letters indicate difference (*p* < 0.05).

Shear force is widely used as an indicator of muscle firmness and overall textural quality in shrimp. The results demonstrated that the control samples had significantly higher shear force values than those of all treated groups (Figure 6). This effect may be associated with extensive denaturation and aggregation of myofibrillar proteins during thermal processing, leading to muscle fiber contraction and water expulsion from the tissue, ultimately resulting in a firmer texture [13,21,33]. This observation is consistent with the findings of Wachirasiri, Wanlapa, Uttapap and Rungsardthong [18], who reported that untreated shrimp tend to develop increased toughness after heat treatment, likely due to enhanced protein aggregation and reduced water retention within the muscle structure [18,21]. In contrast to the control, shrimp subjected to soaking treatments exhibited reduced shear force values. However, further substitution of STPP with KTPP or TKPP did not significantly affect shear force compared to the positive control. This reduction in toughness may be explained by the formation of a water-retaining protein network within the treated muscle, which helps mitigate thermal-induced muscle fiber contraction during cooking. Thus, soaking solutions containing phosphate blends and L-lysine effectively suppress excessive shrinkage of myofibrillar proteins during thermal processing, leading to im-proved textural quality of shrimp muscle [18,41].

### 3.6. Microstructure

**Figure 7:**
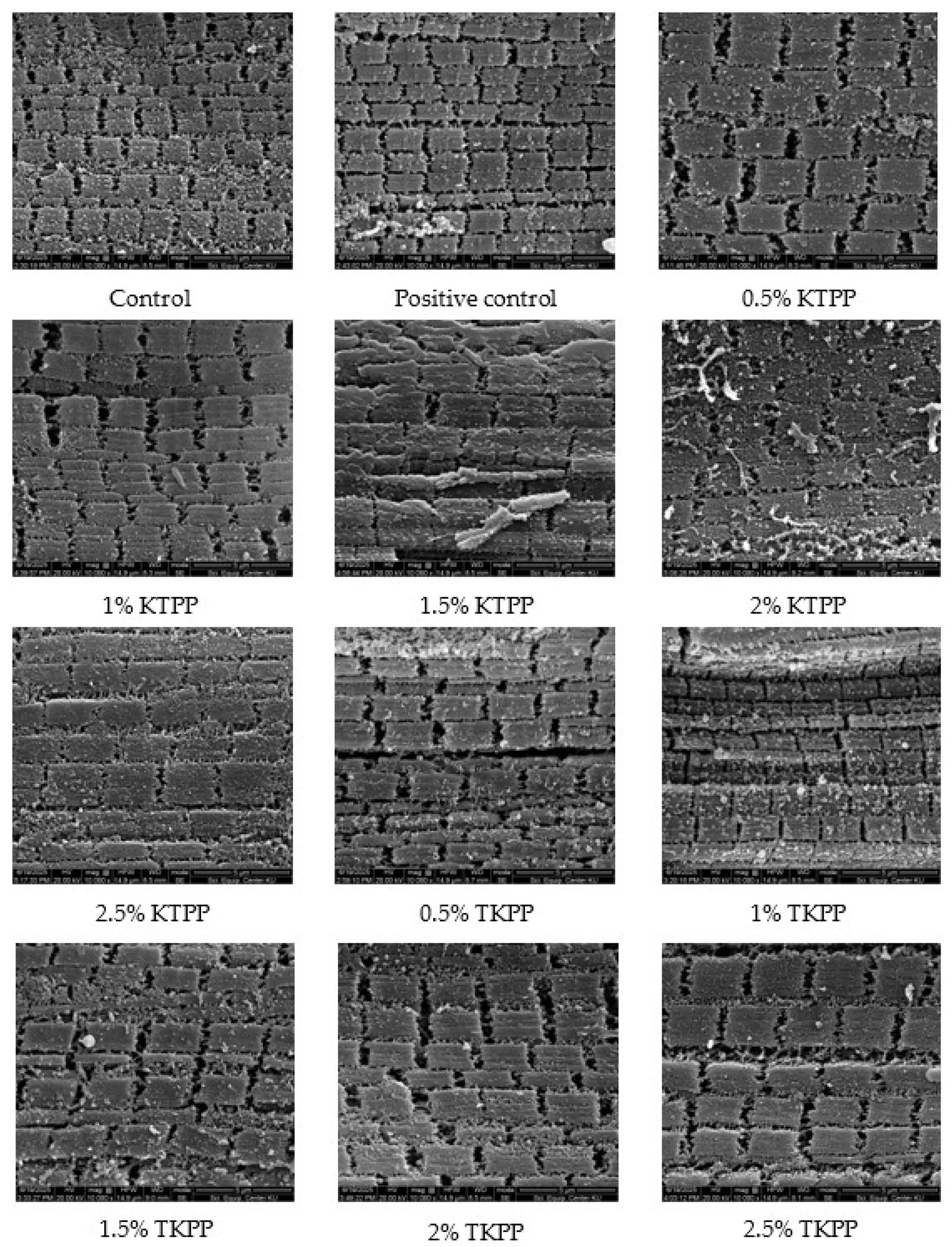
SEM micrographs of longitudinal sections of cooked shrimp muscle treated with different soaking solutions. Magnification = 10,000×. The positive control consisted of 2.5% NaCl, 3.0% sodium tripolyphosphate (STPP), and 2.0% L-lysine. In the potassium tripolyphosphate (KTPP) or tetrapotassium pyrophosphate (TKPP) treatments, STPP was partially replaced with KTPP or TKPP at levels ranging from 0.5 to 2.5% (*w*/*v*).

**Figure 8:**
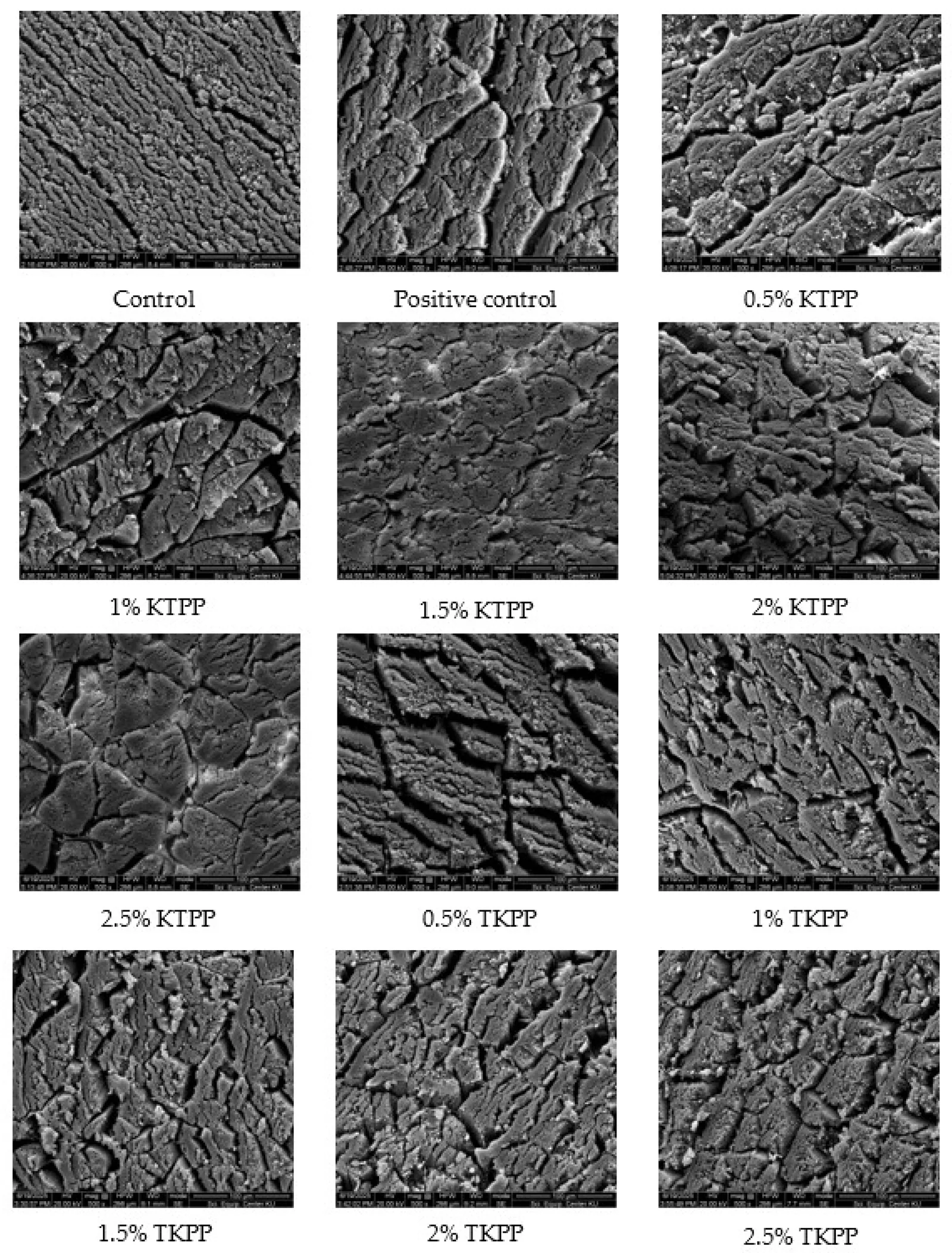
SEM micrographs of transverse sections of cooked shrimp muscle treated with different soaking solutions. Magnification = 500×. The positive control consisted of 2.5% NaCl, 3.0% sodium tripolyphosphate (STPP), and 2.0% L-lysine. In the potassium tripolyphosphate (KTPP) or tetrapotassium pyrophosphate (TKPP) treatments, STPP was partially replaced with KTPP or TKPP at levels ranging from 0.5 to 2.5% (*w*/*v*).

The relationship between muscle microstructure and water-holding capacity (WHC) was qualitatively observed through the expansion of interfilament spaces observed in scanning electron microscopy (SEM) images (Figure 7 and Figure 8). SEM observations indicated that the control samples exhibited a more compact muscle fiber arrangement, whereas treated samples displayed more relaxed and expanded microstructures. These structural differences may be associated with myofibrillar swelling under the alkaline soaking conditions and may contribute to the observed differences in water retention (Figure 2 and Figure 3). Consistent with the findings of Wachirasiri, Wanlapa, Uttapap and Rungsardthong [18], shrimp samples treated with STPP combined with amino acids (lysine and arginine) exhibited noticeable swelling of the myofibrils at the Z-disks. Such microstructural alterations have been reported to accompany myofibrillar expansion and may be related to water retention in shrimp muscle. Notably, partial substitution of STPP with KTPP or TKPP did not further affect the extent of interfilament expansion compared with the positive control, suggesting similar microstructural alterations among the phosphate-blended treatments. This similarity may be related to the effects of the phosphate salts on the pH and ionic environment of the muscle system, which may influence protein charge development and myofibrillar structure [6]. This similarity may be attributed to the combined effects of ionic strength and alkaline conditions in the soaking solutions, which could promote protein charge development and myofibrillar swelling, thereby facilitating water retention within the muscle matrix [18,28]. This observation is consistent with the theory proposed by Kingwascharapong and Benjakul [23,42,43], which states that water retention in muscle foods is largely governed by the expansion of the protein matrix at the molecular level. Consequently, partial replacement of STPP with KTPP or TKPP in combination with L-lysine may serve as a promising strategy for sodium reduction while maintaining processing yield and flesh quality in seafood products.

### 3.7. Phosphate Content

**Figure 9:**
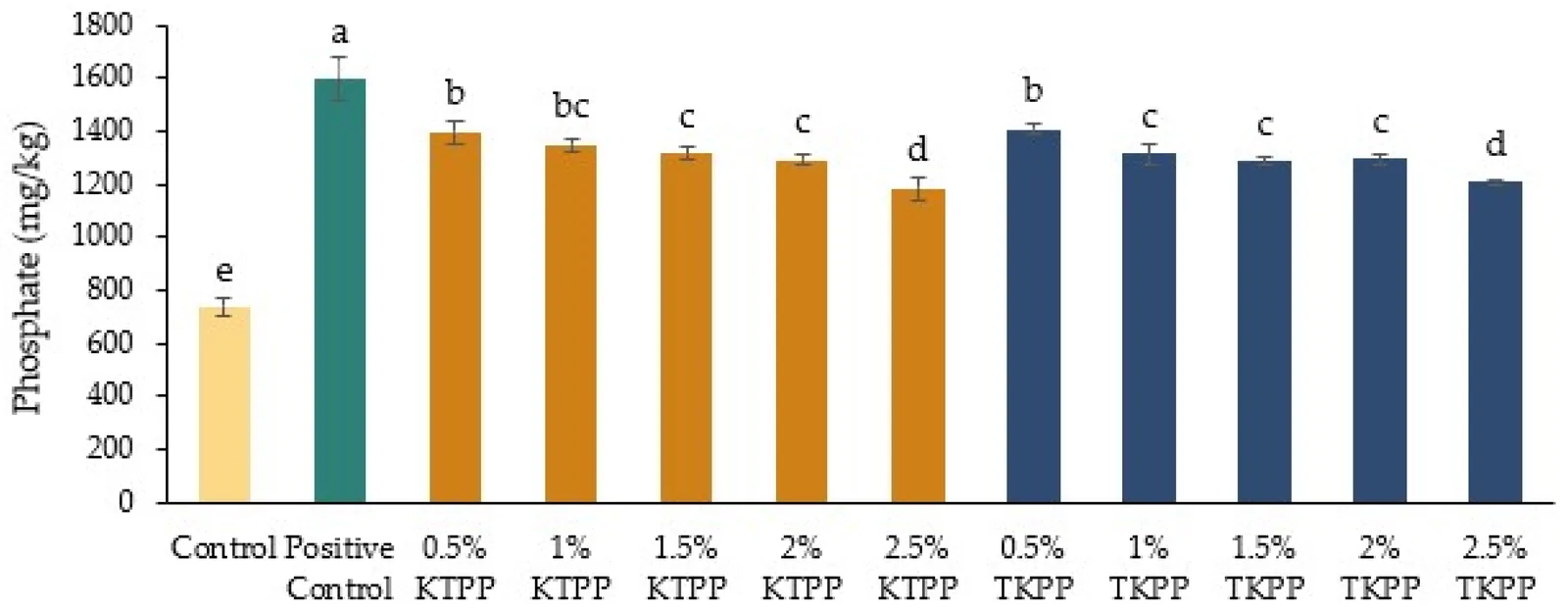
Total phosphate content of cooked shrimp treated with different soaking solutions. The positive control consisted of 2.5% NaCl, 3.0% sodium tripolyphosphate (STPP), and 2.0% L-lysine. In the potassium tripolyphosphate (KTPP) or tetrapotassium pyrophosphate (TKPP) treatments, STPP was partially replaced with KTPP or TKPP at levels ranging from 0.5 to 2.5% (*w*/*v*). Different lowercase letters indicate difference (*p* < 0.05).

The total phosphate levels detected in the cooked shrimp samples are illustrated in Figure 9. The positive control, containing 100% STPP, exhibited the highest phosphate accumulation (1598 mg/kg). However, this value remained well below the maximum allowable limit for total phosphates in food products (2200 mg/kg) established by the Thai Ministry of Public Health (Notification No. 418). Accordingly, all treatments complied with national regulatory standards. Interestingly, the total phosphate content in shrimp muscle exhibited a significant decreasing trend as the proportion of STPP replaced with sodium-free phosphates (KTPP or TKPP) increased. This reduction was consistent with the phosphorus distribution detected by EDX analysis (Figure S1a). The observed decrease could be attributed to the inherent chemical characteristics of the phosphate salts [44]. The decrease can be attributed to differences in the intrinsic phosphorus content of the phosphate salts. As reported by Thangavelu, Kerry, Tiwari and McDonnell [29], STPP contains a higher phosphorus pentoxide (P_2_O_5_) content (57.9%) compared to KTPP (47.5%) and TKPP (43.0%). This difference in phosphorus content may partly contribute to the lower total phosphate levels observed in shrimp muscle following substitution with KTPP or TKPP. In addition, the use of mixed phosphate systems may potentially lead to competitive interactions among phosphate species during diffusion and binding within the muscle matrix. Differences in ionic radius and hydration properties between sodium and potassium ions could play a role in influencing the penetration efficiency and affinity of phosphate ions toward myofibrillar proteins [32]. Furthermore, the presence of potassium-based phosphates might alter the ionic strength and charge distribution in the soaking solution, which may, in turn, affect the binding capacity of phosphate groups to protein sites [28]. Notably, the highest substitution level (2.5%) of KTPP or TKPP resulted in up to a 25% reduction in total phosphate content relative to the positive control, highlighting a potential nutritional benefit alongside sodium reduction.

### 3.8. Sodium and Potassium Content

**Figure 10:**
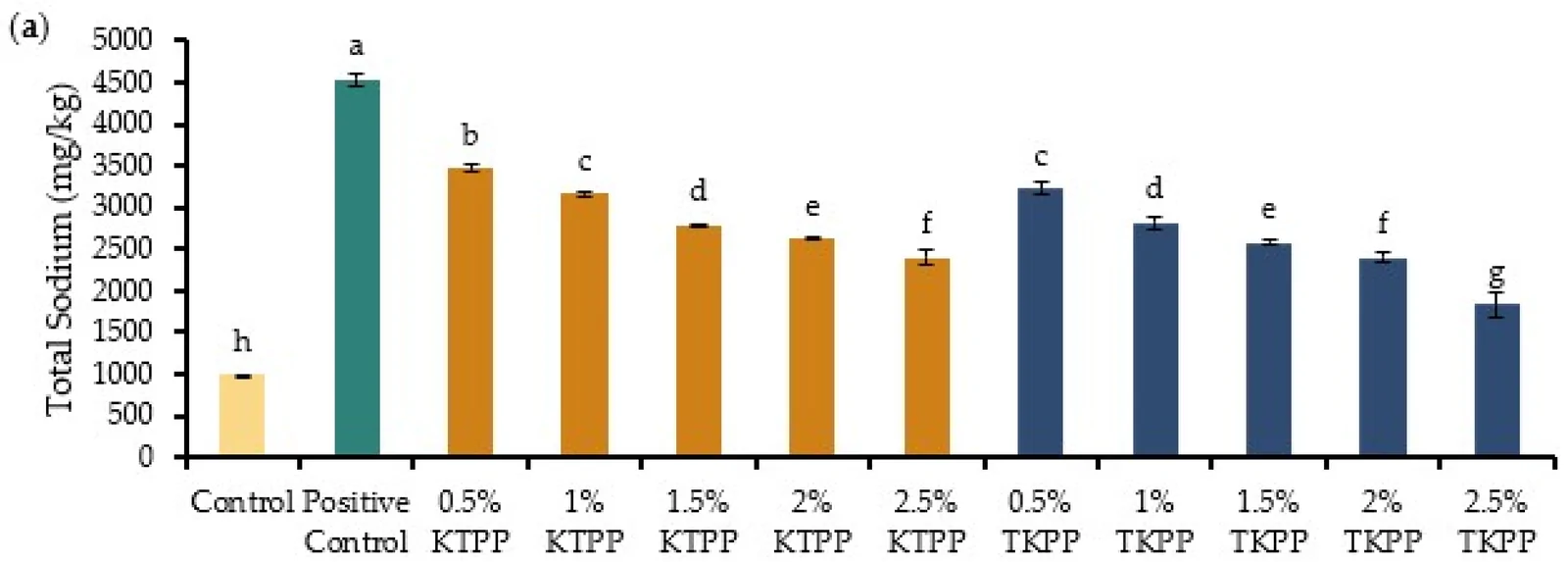

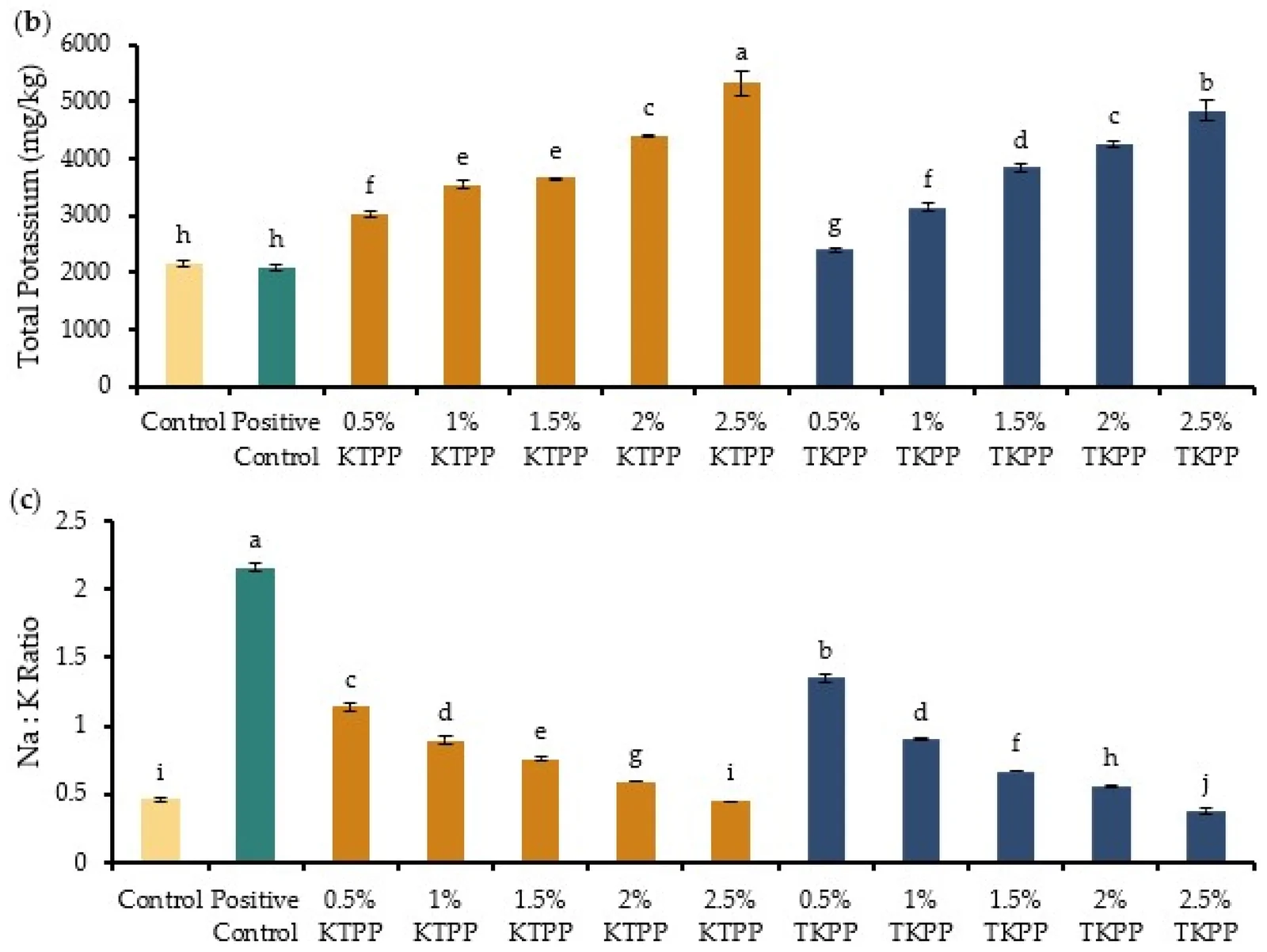
Sodium content (**a**), potassium content (**b**), and sodium–potassium (**c**) ratio of cooked shrimp treated with different soaking solutions. The positive control consisted of 2.5% NaCl, 3.0% sodium tripolyphosphate (STPP), and 2.0% L-lysine. In the KTPP and TKPP treatments, STPP was partially replaced with potassium tripolyphosphate (KTPP) or tetrapotassium pyrophosphate (TKPP) at levels ranging from 0.5 to 2.5% (*w*/*v*). Different lowercase letters indicate difference (*p* < 0.05).

Figure 10 illustrates the sodium and potassium contents, as well as the sodium-to-potassium ratio, in the cooked shrimp samples. Mineral composition analysis showed that the positive control group exhibited the highest sodium level. The partial substitution of STPP with KTPP or TKPP resulted in a progressive decrease in sodium levels in the cooked shrimp. Specifically, sodium content was reduced by 23.5–47% with KTPP and by 28.8–59.5% with TKPP. At equivalent concentrations, TKPP demonstrated a significantly greater sodium-reduction efficiency than KTPP (Figure 10a). These results align with the findings of Desmond [6], who reported that potassium-based phosphates are effective alternatives for sodium reduction while maintaining key functional properties such as water-binding capacity and ionic strength. Notably, all treatments involving partial substitution of STPP with KTPP or TKPP, except for the 0.5% KTPP treatment, met the criteria for a “reduced sodium” labeling claim established by the Thai Food and Drug Administration (Thai FDA), which requires a reduction of at least 25% compared with the reference food [45]. This trend was further supported by EDX mapping, which confirmed a decrease in sodium signal intensity in samples treated with potassium-based phosphates (Figure S1b). In contrast, the use of potassium-based phosphates significantly increased the potassium content of cooked shrimp. At comparable substitution levels, KTPP treatments resulted in significantly higher potassium enrichment than TKPP. Specifically, potassium content increased by 155.1% and 131.8% in the 2.5% KTPP and 2.5% TKPP treatments, respectively, compared with the positive control (Figure 10b). A corresponding increase in potassium signal intensity was also observed in EDX mapping (Figure S1c). According to recommendations from the Thai Recommended Daily Intakes (Thai RDIs), the recommended daily intake of potassium for adults is approximately 3500 mg. The results suggest that replacing STPP with KTPP or TKPP produced potassium-enriched shrimp products. Higher dietary potassium intake has been associated with reduced blood pressure, a decreased risk of kidney stone formation, and a potential reduction in bone loss [46]. Consequently, the changes in sodium and potassium contents significantly reduced the Na/K ratio, as illustrated in Figure 10c. Increasing the level of STPP replacement progressively lowered the Na/K ratio, with the positive control exhibiting the highest value and the 2.5% TKPP treatment the lowest. This reduction highlights an important nutritional advantage, as a lower Na/K ratio has been associated with reduced risk of hypertension and cardiovascular disease [47].

### 3.9. Electronic Tongue

**Figure 11:**
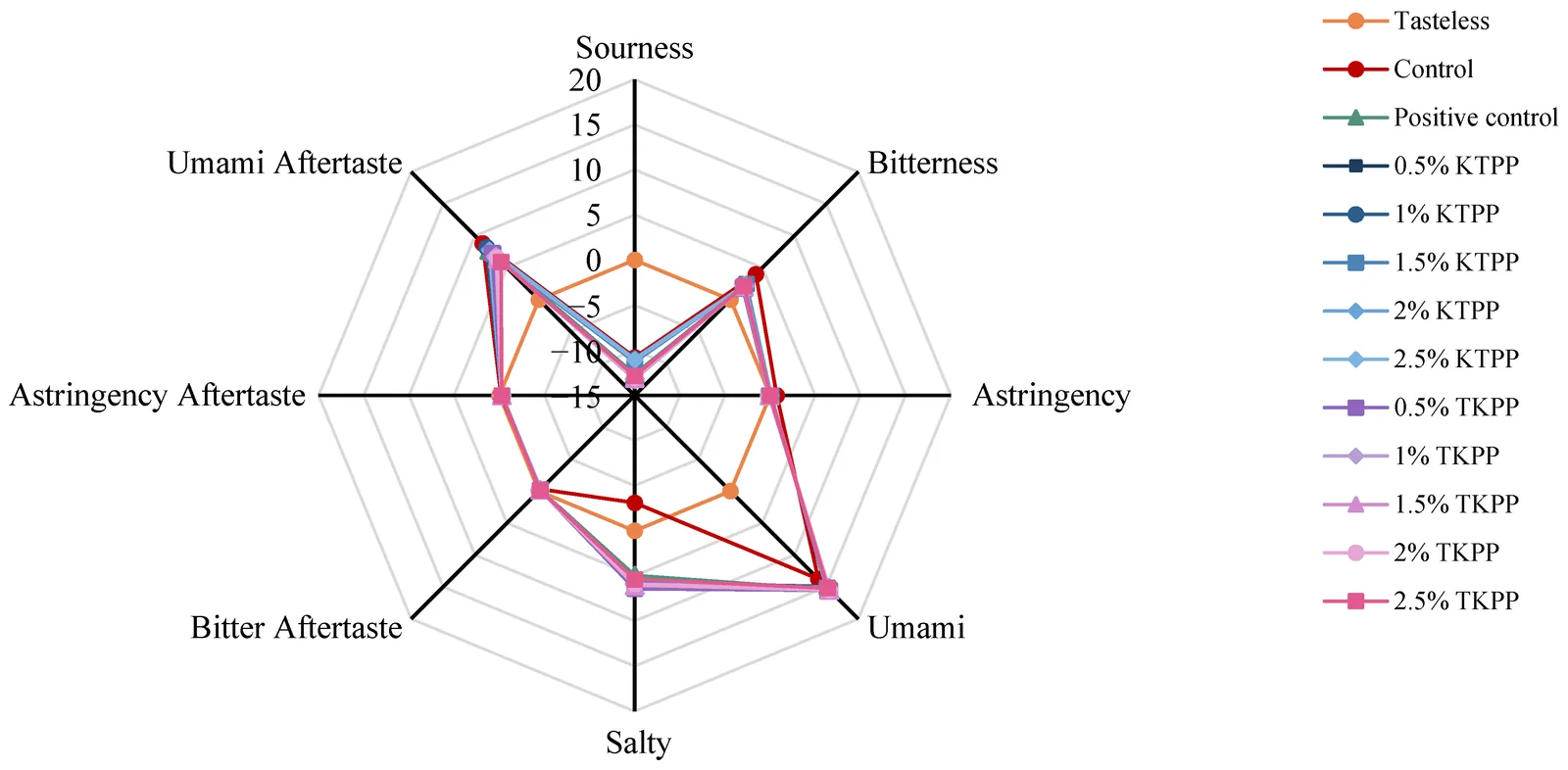
E-tongue radar plot of cooked shrimp treated with different soaking solutions. The scale value (from −15 to 20) indicates that the response value of the sensor is equivalent to the level of taste value. The positive control consisted of 2.5% NaCl, 3.0% sodium tripolyphosphate (STPP), and 2.0% L-lysine. In the potassium tripolyphosphate (KTPP) or tetrapotassium pyrophosphate (TKPP) treatments, STPP was partially replaced with KTPP or TKPP at levels ranging from 0.5 to 2.5% (*w*/*v*).

The electronic tongue uses chemical sensor arrays to characterize taste-related responses and objectively evaluate multiple taste attributes [48]. The taste profiles of the cooked shrimp are presented in Figure 11. Radar plot analysis indicated that umami, saltiness, and bitterness were the predominant taste attributes across all samples. The control sample (red line) exhibited a distinct taste profile, characterized by lower saltiness and slightly higher bitterness compared with the treated samples. In contrast, both the positive control and treated samples showed higher instrumental sensor responses for saltiness and umami, along with reduced bitterness. These differences in instrumental sensor responses may be associated with the presence of sodium and L-lysine in the soaking solutions. Lysine has been reported to act as a bitterness-masking agent while enhancing umami perception, possibly through interactions with taste receptors associated with umami perception and suppression of bitter taste responses [13]. The E-tongue response for sourness was below the zero-reference point, suggesting a relatively low intensity of this attribute. Thus, partial substitution of STPP with KTPP or TKPP did not markedly affect the overall instrumental taste profile of the shrimp samples.

### 3.10. Preliminary Consumer Test

The consumer acceptance scores of cooked shrimp subjected to different soaking treatments are presented in Table 4. In terms of appearance, the control sample received the lowest score among all treatments. This may be attributed to its relatively firmer and less hydrated appearance compared with the treated samples, which likely reduced its visual acceptability. This observation is consistent with the texture scores, where the control sample also received the lowest rating, indicating a firmer and less desirable texture compared with the treated samples. Such differences may be explained by the improved water-holding capacity in the treated samples, where soaking in phosphate- and lysine-containing solutions promotes protein hydration and myofibrillar swelling, resulting in a more tender and juicier texture.

In terms of flavor, the positive control received the highest score among all samples. A decreasing trend in flavor scores was observed with increasing levels of STPP substitution by KTPP or TKPP. This decline may be attributed to the development of bitter and astringent off-flavors associated with potassium-based salts, particularly at higher substitution levels, where the intensity of these off-flavors may have become more pronounced, particularly in samples treated with 2.0–2.5% KTPP or TKPP.

In terms of overall acceptability, the control sample received a relatively high score (7.10). This may be explained by consumers’ familiarity with the typical characteristics of non-treated shrimp, which could influence their preference despite its relatively firmer texture.

In addition, overall acceptability is influenced by multiple factors, including individual preferences and prior experience, which may contribute to variability in consumers’ responses [18,21,30]. As expected, the positive control achieved the highest overall acceptance. In contrast, samples treated with KTPP or TKPP generally showed lower scores, particularly at higher substitution levels. This reduction may be partly associated with the development of slight off-flavors, including bitter and metallic aftertastes, which have been reported as potential sensory limitations of potassium-based salts used in sodium-reduced food formulations [6,49]. Although some consumers were able to perceive these off-notes, samples treated with higher levels of potassium phosphates still exhibited relatively high consumer acceptance scores (>7) in several attributes. Notably, the 1% KTPP treatment received the highest appearance score (7.10), while its flavor (7.90) and overall acceptability (7.70) scores remained relatively high and were not significantly different from those of several other phosphate-substituted treatments (Table 4). In contrast, the highest color score was observed for the 2% TKPP treatment (7.90), indicating that consumer responses varied depending on the evaluated attribute. Overall, the potassium phosphate-treated samples generally maintained acceptable sensory characteristics, although higher substitution levels tended to be associated with lower flavor and overall acceptability scores. These findings suggest that partial substitution of STPP with KTPP or TKPP can be achieved without markedly compromising consumer acceptance.

## 4. Conclusions

This study investigated the effects of substituting STPP with varying proportions of KTPP and TKPP, in combination with L-lysine, on the physicochemical properties, microstructure, and consumer acceptance of PWS. The results indicated that increasing proportions of KTPP or TKPP gradually decreased weight gain and resulted in variations in pH and color parameters, with some differences being statistically significant among formulations. However, partial replacement of STPP with these alternatives at low-to-moderate levels had no significant effect on cooking yield or water-holding capacity (WHC). Microstructural observations revealed a more expanded muscle matrix, indicating myofibrillar swelling, which may be associated with water retention and the observed lower shear force. However, increasing substitution levels did not significantly alter shear force compared with the positive control. In addition, substitution with KTPP or TKPP reduced phosphate and sodium contents. Sodium levels decreased by 23.5–47.0% with KTPP and 28.8–59.5% with TKPP, resulting in a lower Na/K ratio and the development of potassium-enriched shrimp products. Electronic-tongue analysis indicated that saltiness and umami were predominant taste attributes across the samples, with phosphate-treated samples generally exhibiting higher instrumental sensor responses for saltiness and umami and lower bitterness than the control. However, partial substitution of STPP with KTPP or TKPP did not markedly alter the overall instrumental taste profile. Consumer acceptance exhibited concentration-dependent trends; higher substitution levels (>1.5%) resulted in lower flavor and overall acceptability scores, which may be associated with potassium-related off-flavors. Among the tested formulations, 1.5% TKPP was considered a promising formulation because it maintained acceptable processing yield, WHC, and consumer acceptance while achieving approximately 43% sodium reduction. However, the present study was limited to a laboratory-scale comparison of phosphate-based soaking treatments under controlled conditions, and the preliminary consumer test involved only 30 consumers, representing a relatively small sample size. Further studies using independent shrimp batches, larger-scale processing conditions, and a larger and more representative consumer population are needed to validate consumer acceptability and the practical applicability of the proposed formulation.

## Figures and Tables

**Table 1:** Composition and proportions of soaking solutions for sodium reduction in PWS, featuring partial replacement of STPP with KTPP or TKPP in the presence of L-lysine (%, *w*/*v*).

Treatments	NaCl	KCl	STPP	KTPP	TKPP	Lysine
Control (non-soaking)	-	-	-	-	-	-
Positive Control	2.5	-	3	-	-	2
0.5% KTPP	1.75	0.75	2.5	0.5	-	2
1.0% KTPP	1.75	0.75	2	1	-	2
1.5% KTPP	1.75	0.75	1.5	1.5	-	2
2.0% KTPP	1.75	0.75	1	2	-	2
2.5% KTPP	1.75	0.75	0.5	2.5	-	2
0.5% TKPP	1.75	0.75	2.5	-	0.5	2
1.0% TKPP	1.75	0.75	2	-	1	2
1.5% TKPP	1.75	0.75	1.5	-	1.5	2
2.0% TKPP	1.75	0.75	1	-	2	2
2.5% TKPP	1.75	0.75	0.5	-	2.5	2

Note: Sodium chloride (NaCl), potassium chloride (KCl), sodium tripolyphosphate (STPP), potassium tripolyphosphate (KTPP), and tetrapotassium pyrophosphate (TKPP).

**Table 2:** pH values of soaking solutions and raw and cooked shrimp treated with different soaking solutions.

Treatments	pH of the Solution		pH of Shrimp	
	Before Soaking	After Soaking	After Soaking	Cooked Shrimp
Control (non-soaking)	-	-	6.63 ± 0.01 ^h^	6.80 ± 0.01 ^h^
Positive Control	7.99 ± 0.00 ^f^	7.93 ± 0.00 ^f^	6.82 ± 0.01 ^e^	6.88 ± 0.01 ^g^
0.5% KTPP	8.00 ± 0.00 ^f^	7.93 ± 0.01 ^f^	7.01 ± 0.04 ^a^	7.14 ± 0.00 ^ab^
1.0% KTPP	7.95 ± 0.00 ^g^	7.88 ± 0.00 ^g^	6.71 ± 0.01 ^g^	7.06 ± 0.00 ^c^
1.5% KTPP	7.94 ± 0.00 ^h^	7.85 ± 0.00 ^h^	6.87 ± 0.02 ^d^	7.14 ± 0.01 ^a^
2.0% KTPP	7.90 ± 0.00 ^i^	7.82 ± 0.00 ^i^	6.90 ± 0.02 ^c^	7.03 ± 0.00 ^d^
2.5% KTPP	7.87 ± 0.00 ^j^	7.78 ± 0.02 ^j^	6.84 ± 0.01 ^e^	7.07 ± 0.01 ^c^
0.5% TKPP	8.17 ± 0.00 ^e^	8.05 ± 0.01 ^e^	6.71 ± 0.01 ^g^	6.78 ± 0.01 ^i^
1.0% TKPP	8.27 ± 0.00 ^d^	8.18 ± 0.00 ^d^	6.75 ± 0.01 ^f^	6.91 ± 0.00 ^f^
1.5% TKPP	8.37 ± 0.00 ^c^	8.24 ± 0.00 ^c^	7.01 ± 0.01 ^a^	7.13 ± 0.00 ^b^
2.0% TKPP	8.43 ± 0.00 ^b^	8.35 ± 0.00 ^b^	6.75 ± 0.02 ^f^	7.00 ± 0.01 ^e^
2.5% TKPP	8.51 ± 0.00 ^a^	8.37 ± 0.00 ^a^	6.97 ± 0.01 ^b^	7.07 ± 0.01 ^c^

Values are given as mean ± SD. Different superscripts in a column indicate differences (*p* < 0.05). The positive control consisted of 2.5% NaCl, 3.0% sodium tripolyphosphate (STPP), and 2.0% L-lysine. In the potassium tripolyphosphate (KTPP) or tetrapotassium pyrophosphate (TKPP) treatments, STPP was partially replaced with KTPP or TKPP at levels ranging from 0.5 to 2.5% (*w*/*v*).

**Table 3:** Color values (L*, a*, b*) of raw and cooked shrimp treated with different soaking solutions.

Treatments	Raw Shrimp			Cooked Shrimp		
	L*	a*	b*	L*	a*	b*
Control (non-soaking)	40.58 ± 0.81 ^b^	−1.07 ± 0.33 ^ns^	−0.86 ± 0.86 ^a^	71.82 ± 0.56 ^ab^	7.57 ± 1.23 ^a^	16.52 ± 0.96 ^a^
Positive Control	37.97 ± 0.97 ^b^	−0.89 ± 0.36 ^ns^	−2.44 ± 0.71 ^bcd^	70.64 ± 1.06 ^bc^	5.90 ± 0.98 ^ab^	13.21 ± 1.37 ^bc^
0.5% KTPP	40.82 ± 2.23 ^b^	−0.92 ± 0.38 ^ns^	−2.06 ± 0.81 ^abc^	70.09 ± 0.57 ^c^	5.26 ± 0.29 ^b^	12.88 ± 0.44 ^bc^
1.0% KTPP	39.65 ± 0.67 ^ab^	−0.96 ± 0.41 ^ns^	−2.24 ± 0.79 ^bcd^	69.95 ± 0.41 ^c^	6.74 ± 0.33 ^ab^	14.56 ± 0.62 ^ab^
1.5% KTPP	39.54 ± 0.48 ^ab^	−0.95 ± 1.46 ^ns^	−2.44 ± 0.76 ^bcd^	70.44 ± 0.82 ^c^	6.98 ± 0.13 ^ab^	14.29 ± 0.22 ^ab^
2.0% KTPP	40.69 ± 0.91 ^b^	−0.86 ± 0.33 ^ns^	−1.96 ± 0.81 ^ab^	69.97 ± 0.83 ^c^	5.27 ± 1.90 ^b^	12.87 ± 3.16 ^bc^
2.5% KTPP	39.92 ± 0.92 ^ab^	−1.24 ± 0.16 ^ns^	−3.11 ± 0.53 ^bcd^	70.07 ± 0.39 ^c^	5.13 ± 0.23 ^b^	11.11 ± 1.89 ^c^
0.5% TKPP	39.47 ± 1.13 ^ab^	−1.19 ± 0.21 ^ns^	−3.35 ± 0.68 ^cd^	70.83 ± 0.47 ^bc^	6.09 ± 1.58 ^ab^	13.26 ± 2.60 ^bc^
1.0% TKPP	40.99 ± 1.94 ^b^	−1.16 ± 0.18 ^ns^	−3.53 ± 0.56 ^d^	70.96 ± 0.97 ^bc^	5.08 ± 0.68 ^b^	11.48 ± 1.21 ^bc^
1.5% TKPP	39.56 ± 1.16 ^ab^	−1.02 ± 0.28 ^ns^	−3.34 ± 0.44 ^cd^	69.75 ± 1.00 ^c^	6.51 ± 1.28 ^ab^	13.25 ± 1.93 ^bc^
2.0% TKPP	40.04 ± 0.11 ^ab^	−1.27 ± 0.93 ^ns^	−3.03 ± 0.74 ^bcd^	72.29 ± 0.08 ^a^	5.23 ± 0.45 ^b^	10.36 ± 0.53 ^c^
2.5% TKPP	39.93 ± 1.27 ^ab^	−0.82 ± 0.45 ^ns^	−2.10 ± 0.23 ^abc^	70.50 ± 0.95 ^c^	5.55 ± 0.86 ^b^	11.59 ± 1.48 ^bc^

Values are given as mean ± SD. Different superscripts in a column indicate differences (*p* < 0.05). ns indicates no differences (*p* ≥ 0.05) The positive control consisted of 2.5% NaCl, 3.0% sodium tripolyphosphate (STPP), and 2.0% L-lysine. In the potassium tripolyphosphate (KTPP) or tetrapotassium pyrophosphate (TKPP) treatments, STPP was partially replaced with KTPP or TKPP at levels ranging from 0.5 to 2.5% (*w*/*v*).

**Table 4:** Preliminary consumer test results of cooked white shrimp treated with different soaking solutions, evaluated using a 9-point hedonic scale (1 = dislike extremely, 5 = neither like nor dislike, and 9 = like extremely).

Treatments	Appearance	Color	Flavor	Texture	Overall
Control	6.20 ± 1.99 ^b^	7.10 ± 1.60 ^c^	7.20 ± 1.75 ^bcd^	6.80 ± 1.93 ^c^	7.10 ± 1.52 ^cde^
Positive Control	7.00 ± 1.76 ^ab^	7.40 ± 1.84 ^ab^	8.00 ± 1.05 ^a^	7.90 ± 1.10 ^a^	8.00 ± 1.15 ^a^
0.5% KTPP	6.80 ± 2.35 ^ab^	7.20 ± 2.15 ^ab^	7.90 ± 0.99 ^ab^	7. 50 ± 1.51 ^abc^	7.90 ± 0.99 ^a^
1% KTPP	7.10 ± 1.29 ^a^	7.50 ± 1.51 ^ab^	7.90 ± 1.20 ^ab^	7.40 ± 1.65 ^abc^	7.70 ± 1.25 ^ab^
1.5% KTPP	6.70 ± 1.70 ^ab^	7.40 ± 1.58 ^ab^	7.50 ± 0.97 ^abcd^	7.30 ± 1.02 ^abc^	7.00 ± 1.41 ^de^
2% KTPP	6.60 ± 1.96 ^ab^	7.20 ± 2.04 ^ab^	6.40 ± 2.32 ^e^	7.30 ± 1.64 ^abc^	7.00 ± 1.70 ^de^
2.5% KTPP	6.70 ± 1.83 ^ab^	7.70 ± 1.34 ^ab^	6.90 ± 1.97 ^de^	7.00 ± 2.54 ^bc^	7.10 ± 1.85 ^cde^
0.5% TKPP	6.30 ± 1.89 ^ab^	7.10 ± 1.6 ^b^	7.50 ± 1.35 ^abcd^	7.40 ± 1.43 ^abc^	7.20 ± 1.14 ^bcde^
1% TKPP	6.60 ± 1.35 ^ab^	7.10 ± 0.99 ^b^	7.30 ± 1.34 ^abcd^	7.50 ± 1.43 ^abc^	7.50 ± 1.08 ^abcd^
1.5% TKPP	6.60 ± 2.12 ^ab^	7.50 ± 1.72 ^ab^	7.70 ± 0.95 ^abc^	7.60 ± 0.93 ^ab^	7.60 ± 0.97 ^abc^
2% TKPP	6.60 ± 1.96 ^ab^	7.90 ± 1.20 ^a^	7.00 ± 1.41 ^cde^	7.30 ± 1.49 ^abc^	7.10 ± 1.20 ^cde^
2.5% TKPP	6.60 ± 1.01 ^ab^	7.50 ± 0.97 ^ab^	6.50 ± 1.51 ^de^	7.40 ± 1.26 ^abc^	6.80 ± 1.23 ^e^

Values are given as mean ± SD. Different superscripts in a column indicate differences (*p* < 0.05). The positive control consisted of 2.5% NaCl, 3.0% sodium tripolyphosphate (STPP), and 2.0% L-lysine. In the potassium tripolyphosphate (KTPP) or tetrapotassium pyrophosphate (TKPP) treatments, STPP was partially replaced with KTPP or TKPP at levels ranging from 0.5 to 2.5% (*w*/*v*).

## Data Availability

The original contributions presented in this study are included in the article/Supplementary Materials. Further inquiries can be directed to the corresponding author.

## Supplementary Materials

The following supporting information can be downloaded at https://www.mdpi.com/article/10.3390/foods15173063/s1, Figure S1: EDX mapping images of phosphorus (a), sodium (b) and potassium (c) of cooked shrimp treated with different substitution ratios of STPP with KTPP or TKPP.
