# Supplementary material for: Development of a Low-Sodium Soaking Solution for Shrimp Processing Using Potassium Phosphates (KTPP and TKPP) and L-Lysine: Impacts on Quality and Water-Holding Capacity
Source: Foods. 2026 Aug 29;15(17):3063. doi: 10.3390/foods15173063 (PMC13565149; doi:10.3390/foods15173063)
Supplement: Supplementary file 1 [file foods-15-03063-s001.zip › foods-4484598-supplementary.pdf]

(a)

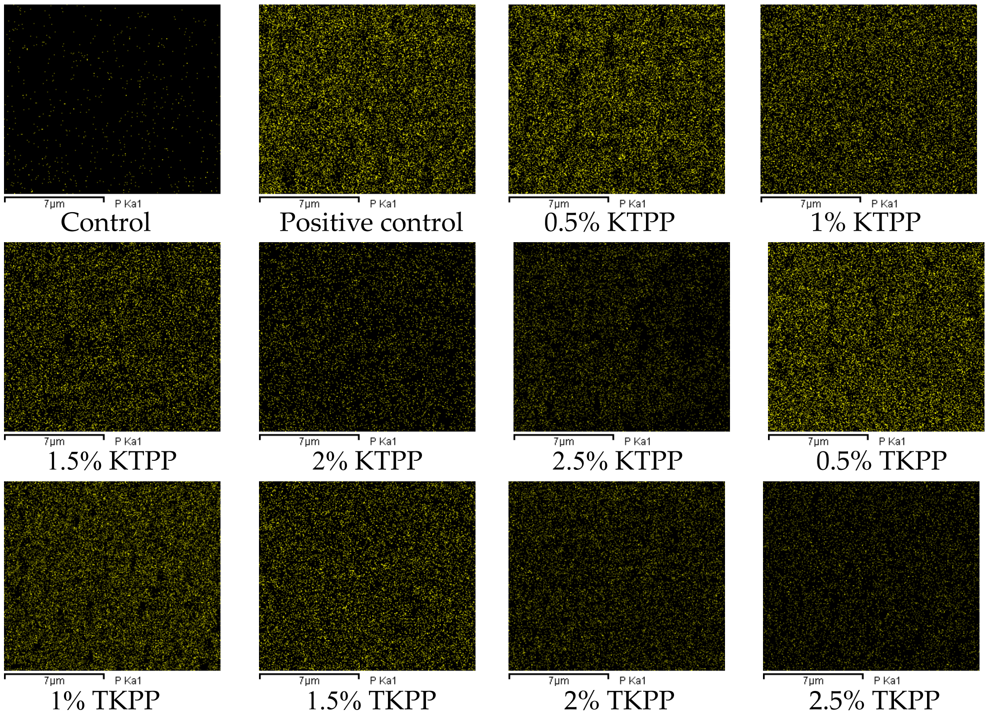
7μm P Kα1
Control
Positive control
0.5% KTPP
1% KTPP
1.5% KTPP
2% KTPP
2.5% KTPP
0.5% TKPP
1% TKPP
1.5% TKPP
2% TKPP
2.5% TKPP

(b)

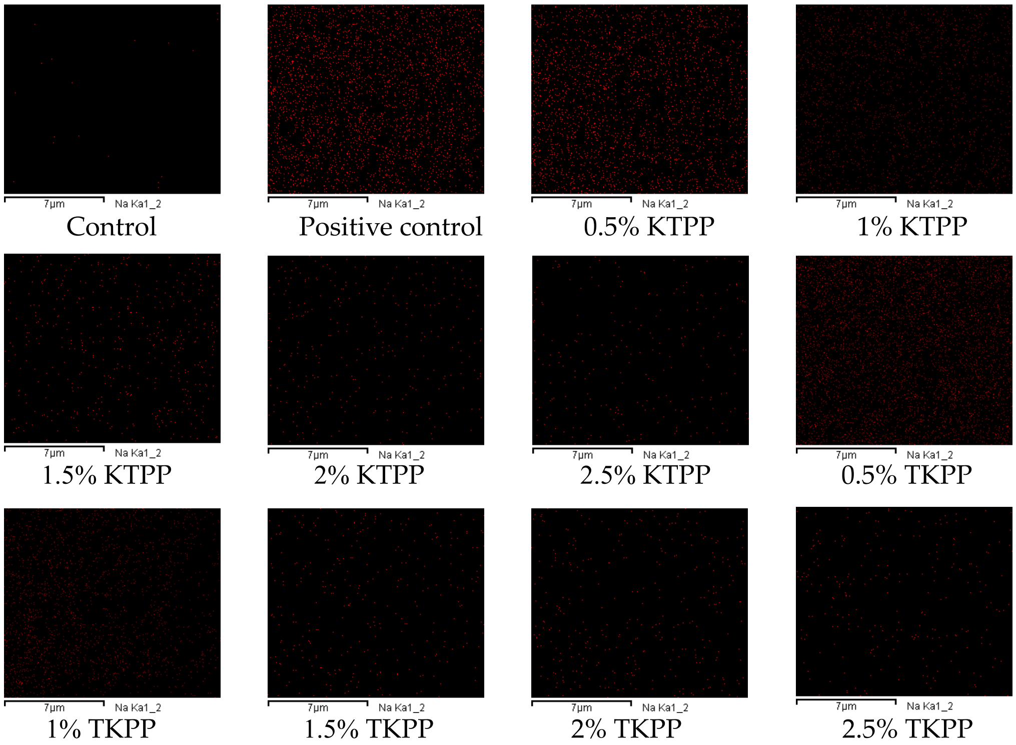
7μm Na Kα1_2
Control
Positive control
0.5% KTPP
1% KTPP
1.5% KTPP
2% KTPP
2.5% KTPP
0.5% TKPP
1% TKPP
1.5% TKPP
2% TKPP
2.5% TKPP

(**c**)

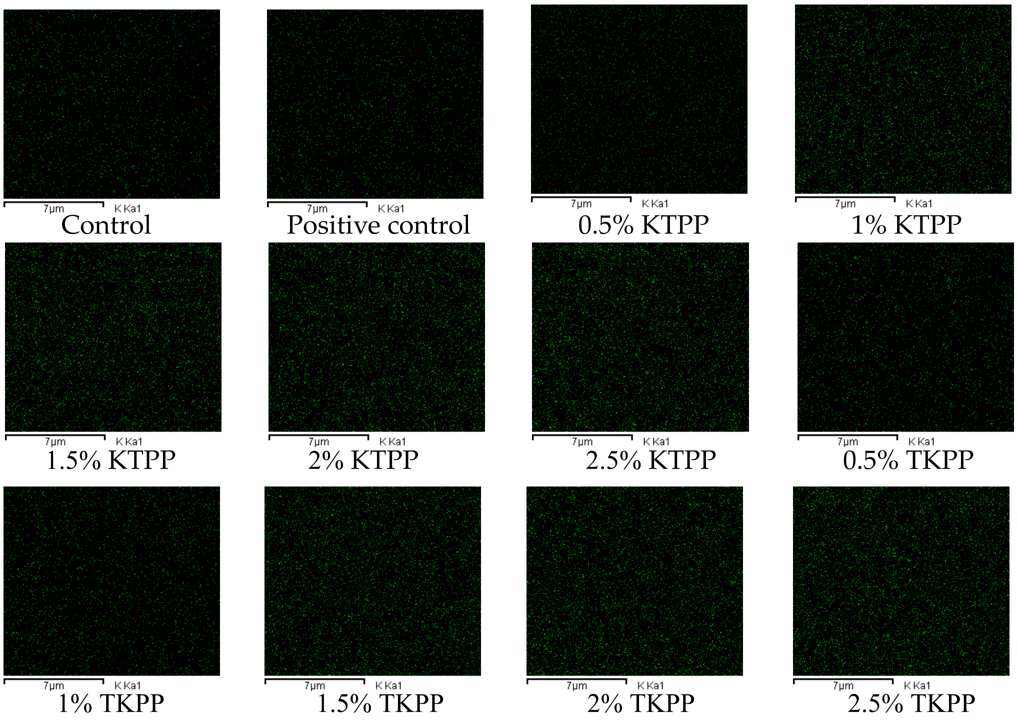

Figure S1. EDX mapping images of phosphorus (a), sodium (b) and potassium (c) of cooked shrimp treated with different substitution ratios of STPP with KTPP or TKPP.
